# Computational Modeling of T Cell Hypersensitivity during Coronavirus Infections Leading to Autoimmunity and Lethality

**DOI:** 10.1155/2022/9444502

**Published:** 2022-03-22

**Authors:** Alper Tunga Celebi, Goksu Uzel, Ece Oylumlu, Cengiz Baykasoglu, Ata Mugan, Sophie Joly, Ceren Ciraci

**Affiliations:** ^1^Vienna University of Technology, Institute of Applied Physics, Wiedner Hauptstrasse 8-10/E134, 1040 Vienna, Austria; ^2^Istanbul Technical University, College of Science and Letters, Molecular Biology and Genetics Department, Istanbul Technical University, Istanbul 34469, Turkey; ^3^Faculty of Engineering Mechanical Engineering Department, Hitit University, 19030 Çorum, Turkey; ^4^Faculty of Mechanical Engineering, Mechanical Engineering Department, Istanbul Technical University, 34437 Istanbul, TR, Turkey; ^5^Biology Department, Kirkwood Community College, Cedar Rapids 52404, USA

## Abstract

The human angiotensin-converting enzyme 2 (hACE2) receptor is the primary receptor for SARS-CoV-2 infection. However, the presence of alternative receptors such as the transmembrane glycoprotein CD147 has been proposed as a potential route for SARS-CoV-2 infection. The outcomes of SARS-CoV-2 spike protein binding to receptors have been shown to vary among individuals. Additionally, some patients infected with SARS-CoV-2 develop autoimmune responses. Given that CD147 is involved in the hyperactivation of memory T cells resulting in autoimmunity, we investigated the interaction of the SARS-CoV-2 viral spike protein with CD147 receptor and retinal specific CD147 Ig0 domain in silico using molecular docking and molecular dynamics (MD) simulations. The results indicated that binding involves two critical residues Lys63 and Asp65 in a ubiquitous CD147 isoform, potentially leading to the hyperactivation of T cells for only SARS-CoV-2, but not for SARS-CoV or MERS-CoV. Overall binding was confirmed by docking simulations. Next, MD analyses were completed to verify the docking poses. Polar interactions suggested that the interaction via Lys63 and Asp65 might be one of the determinants associated with severe COVID-19 outcomes. Neither did SARS-CoV nor MERS-CoV bind to these two critical residues when molecular docking analyses were performed. Interestingly, SARS-CoV was able to bind to CD147 with a lower affinity (-4.5 kcal/mol) than SARS-CoV-2 (-5.6 kcal/mol). Furthermore, Delta and Omicron variants of SARS-CoV-2 did not affect the polar interactions with Lys63 and Asp65 in CD147. This study further strengthens the link between SARS-CoV-2 infection and autoimmune responses and provides novel insights for prudent antiviral drug designs for COVID-19 treatment that have implications in the prevention of T cell hyperactivation.

## 1. Introduction

Severe acute respiratory syndrome (SARS) coronavirus (CoV) 2, the pathogen confirmed to be responsible for COVID-19, has had dreadful effects around the world. The unprecedented emergence of the ongoing pandemic entailed extensive research focusing on the SARS-CoV-2 pathogenesis and host-virus interactions. Numerous studies have shown that the angiotensin-converting enzyme 2 (ACE2) receptor mediates the SARS-CoV-2 spike protein binding to human cells [1, 2]. Coronaviruses responsible for SARS and MERS outbreaks are closely related to SARS-CoV-2, and their binding to multiple host receptors via their spike protein suggested the presence of different binding partners [3, 4]. Interestingly, an additional receptor, Cluster of Differentiation 147 (CD147), also known as Basigin, EMMPRIN, has been proposed to bind SARS-CoV-2 in humans [5]. However, current literature presents conflicting data that make the interaction of SARS-CoV-2 spike protein and CD147 a controversial research subject that remains to be elucidated [5–7].

CD147 is a transmembrane glycoprotein of the immunoglobulin superfamily shown to play roles in a wide range of physiological and pathological processes including wound healing, inflammation, microbial infections, tumor cell metastasis, and developmental processes. Its gene expression in white blood cells, platelets, and endothelial cells might be the reason for its broad functionality [8–10]. In particular, knowing the fact that CD147 promotes HIV-1 infection through the interaction with virus-associated cyclophilin [11], therefore, we sought to elucidate the possibility that SARS-CoV-2 uses CD147 to invade host cells and to identify the specific partners in the ligand-receptor interaction.

Four CD147 isoforms are expressed in humans, and their numbers of extracellular Ig-like domains vary [10]. Both of the isoforms have an extracellular region, a transmembrane region, and a cytoplasmic tail. Differential splicing and the transcription initiation site variations give rise to the production of four isoforms. CD147 Ig1-Ig2 isoform carries two extracellular Ig-like domains and is expressed in most tissues. However, the CD147 Ig0-Ig1-Ig2 isoform carries an additional Ig-like domain called Ig0 which is specific to the retina. CD147 ectodomain induces matrix metalloproteinases (MMPs) and cytokines, resulting in inflammation whereas retinal specific CD147 Ig0 is largely unknown [5, 12].

Clinical outcomes of SARS-CoV-2 infection vary among individuals ranging from asymptomatic infections to death. COVID-19 lethality had been associated with hereditary errors of Toll-like receptor 3- (TLR3-) and interferon regulatory factor- (IRF7-) dependent type I interferon (IFN) immunity [13] and, strikingly, the presence of neutralizing IgG autoantibodies against IFN-*ω*, IFN-*α*, or both at the earlier stages of the disease. Type I IFN-related COVID 19 lethality rate was reported as 10% [14].

Failure of the immune system to distinguish self from non-self-antigens results in unintended immune responses, systemic autoimmunity, and tissue damage. Because the immunosuppressive regulatory T cells (Treg) are of great importance for the maintenance of immune homeostasis, defects in their functions are the source of many autoimmune diseases. For example, hyperactive T cells are known to be a key player in the development of systemic lupus erythematosus (SLE); thus, elucidating the underlying mechanism of T cell hyperactivity would be crucial for the discovery of therapeutic targets [15]. Interestingly, a high level of CD147 has been shown to be an indicator for the Treg cell function in several autoimmune diseases [16]. Additionally, in rheumatoid arthritis (RA), one of the most common inflammatory diseases whose mechanism is not fully understood yet, multiple studies have indicated the involvement of CD147 Ig1-Ig2 in the dysregulation of T cells [17–19]. CD147 was initially defined as a T cell activation-associated antigen because of the close association between CD147 and T cell activation and proliferation [20]. Although CD147 expression is low on the resting T cells, it is relatively high in activated T cells [21]. Therefore, participation of CD147 in the pathogenesis of autoimmune diseases is plausible, given that inflammation-related diseases are mediated by abnormal activation of different T cell subsets [9, 22, 23]. In the case of RA and SLE, high expression of CD147 on T lymphocytes in peripheral blood and in local lesions might explain how CD147 plays a role in autoreactive T cell activation. Guo et al. (2019) have shown that two critical residues, Lys63 and Asp65, in CD147 facilitate memory CD4^+^ T cell hyperactivation in RA. In that study, a structural and functional method demonstrated the importance of these epitopes in the interface between 5A12 (anti-CD147 mAb) and CD147. Furthermore, they were able to confirm this finding when the double mutation K63A/D65A inhibited T cell activation in a manner similar to the neutralization by 5A12 [17]. Given that Lys63 and Asp65 are two critical epitopes in CD147 for the hyperactivation of T cells, we modeled the interaction between SARS-CoV-2 and CD147 at the molecular level and investigated whether Lys63 and Asp65 are involved in the binding using molecular docking and molecular dynamics (MD) analyses. Our findings obtained from molecular docking and MD analyses showed that SARS-CoV-2 spike protein open and closed forms bind to CD147 Ig1-Ig2's external domain with a higher affinity than to CD147 Ig0-Ig1-Ig2 (retinal isoform). This result was further explained by the fact that spike protein forms were found to specifically bind to Lys63 and Asp65, two epitopes that are missing in the retinal CD147 isoform. Notably, the binding of spike protein open form had a higher affinity than its closed form. Surprisingly, no binding with Lys63 and Asp65 was observed in any of the molecular docking analyses for Severe acute respiratory syndrome-related coronavirus (SARS-CoV) and the Middle East respiratory syndrome-related coronavirus (MERS-CoV). Moreover, although SARS-CoV was able to bind to CD147 with a lower affinity than SARS-CoV-2, no successful binding results were obtained from docking analysis of MERS-CoV and CD147. Our results provide a theoretical basis for SARS-CoV-2 spike protein binding to CD147's critical epitopes. This particular interaction might explain the hyperactivation of T cells that could lead to the lethality of SARS-CoV-2 infections and also contribute to the development and persistence of an autoimmune response as observed in some of the COVID-19 symptoms of long haulers. Alternatively, CD8^+^ T cells might have a hyperactivation profile with increased cytotoxicity, as they also bear CD147 on their surface. However, such an interaction was not determined in docking analysis of SARS-CoV and CD147. Our results open up the possibility that SARS-CoV-2, but not SARS-CoV, spike protein elicits the development of a hyperactive CD4^+^ T cell state in patients with COVID-19 via a specific interaction with CD147.

## 2. Methods and Algorithms

### 2.1. Molecular Docking

The cryoEM maps and atomic models have been deposited at the Electron Microscopy Data Bank and the Protein Data Bank with accession codes PDB ID: 6VXX (closed SARS-CoV-2 S), as well as PDB ID: 6VYB (SARS-CoV-2 S with one S^B^ open), PDB ID: 5XLR (structure of SARS-CoV spike glycoprotein), and PDB ID: 5X59 (prefusion structure of MERS-CoV spike glycoprotein, threefold symmetry). Crystal structures of SARS-CoV-2 spike protein complex with hACE2 reveal that the virus utilizes the external subdomain of the spike receptor-binding domain (RBD) to recognize the hACE2 receptor [17] and CD147 [5]. As the binding sites of open and closed forms of SARS-CoV-2 spike protein are unknown, complete S proteins were used in in silico docking studies using AutoDock Vina [24]. The open and closed forms of SARS-CoV-2 spike protein docking trials were repeated on four ligand models as follows: binding to (i) complete CD147 receptor, (ii) complete retinal specific CD147 Ig0 domain, (iii) Lys63 residue in CD147 using small and large localized domains, and (iv) Asp65 residue in CD147 using small and large localized domains. Additionally, docking analysis using SARS-CoV and MERS-CoV spike protein binding to CD147 and retinal CD147 was completed. In order to reduce the CPU times, the gridbox in the AutoDock software was set to cover the surroundings of the epitopes Lys63 and Asp65 with the grid dimensions of 15 Å and 50 Å in each direction by centering the Lys63 and Asp65 residues. Thus, small and large localized domains around the epitopes Lys63 and Asp65 were used in AutoDock Vina in the preliminary assessment of molecular interactions with Lys63 and Asp65. These localized domain results confirmed that there were molecular interactions between both the open- and closed-state SARS-CoV-2 and the critical epitopes of Lys63 and Asp65. Unlike the majority of the reported studies, we also used the entire molecules for all receptors and ligands instead of the structural subdomains of these molecules such as the receptor-binding domains. Docking results of complete CD147 receptors gave a better insight into the studied problem. Molecular docking results using small and large localized domains around Lys63 and Asp65 in CD147 were not presented due to limited space.

The performance of ten docking programs was evaluated by examining the accuracies of binding pose prediction (sampling power) and binding affinity estimation (scoring power) [25], wherein it was concluded that the ligand-binding poses could be predicted in most cases by these programs; however, the rankings of binding affinities could not be well predicted by most of them. Moreover, Pagadala et al. [26] reviewed the commercial and academic docking programs and concluded that the capability of identifying the ligand-binding poses by the commercial programs is slightly better than those of the academic programs. Therefore, beforehand, the performance of AutoDock Vina was compared with that of the Haddock web server used for identifying the interactions between hACE2 and open-state SARS-CoV-2 spike protein that are presented in the literature (data not shown) [27, 28]. Then, the binding sites calculated by AutoDock Vina were compared with the atomic model of PDB ID: 6M17 that has been frequently used in silico studies. Binding sites of Autock Vina and Haddock were similar and complied with the PDB ID: 6M17 atomic model while the rankings of binding affinities and residues having interactions could vary. Following, AutoDock Vina docking poses were used as initial predictions in MD analyses.

### 2.2. Protein Preparation

The receptors were initially prepared by removing irrelevant molecules and crystallographic water molecules, adding polar hydrogen molecules, and assigning the Kollman united atom charges using AutoDock Tools version 1.5.6, e.g., see [29, 30]. For the ligands, all hydrogen was added before computing the Gasteiger charges, and then, nonpolar hydrogen was merged. The prepared receptor and ligand structures were saved in the PDBQT file format. Since the binding sites were unknown, the induced-fit docking was initially performed against the receptors to reveal the protein conformational changes in the binding process. The Autogrid was set to cover the entire molecule with the grid spacing of 1 Å and then by adjusting the grid size in *x*, *y*, and *z* directions.

### 2.3. Binding Free Energy Calculation

As docking programs are interested in reproducing chemical potentials determining the bound conformation preference and the free energy of binding, a scoring function is used to approximate the standard chemical potentials of the molecules. The scoring function of AutoDock Vina is a weighted sum of steric interactions, identical for all atom pairs, hydrophobic interaction between hydrophobic atoms, and hydrogen bonding [24].

As shown in an earlier study [31], the efficacy and accuracy of docking were reported to be significantly improved via the inclusion of crystallographic water molecules in the binding site of numerous protein structures. Thus, crystallographic water molecules were expected to improve the accuracy of docking predictions using the scoring function in AutoDock Vina. However, no difference in the docking accuracy was observed, in the presence of water molecules in the vicinity of the ligand. Finally, when a worsening of the docking accuracy was observed [31], this seemed to be due to the inability of the scoring function to account properly for hydrophobic interactions. Additionally, the scoring function of AutoDock Vina is a weighted sum of steric interactions which are identical for all atom pairs and hydrophobic interactions between hydrophobic atoms and hydrogen bonding [24]. In brief, docking analyses were repeated with and without the crystallographic water molecules to ensure the docking modes, and all results are presented in Section 3.

### 2.4. Binding Site Determination

On the determination of binding modes, AutoDock Vina [24] employs iterated local search global optimizer using the nonlinear solver Broyden-Fletcher-Goldfarb-Shanno (BFGS) method for local optimization. It gives the top twenty estimated free energies of binding scores and corresponding RMSD values for each binding mode. It employs the scoring function and its derivative to determine the position and orientation of spike protein molecules, and torsions for the active rotatable bonds of proteins and their flexible residues. Following the determination of binding sites of the proteins, potential binding sites were examined through sequential docking by setting the Autogrid size to particular regions of the receptor with the selected grid spacing. SARS-CoV, MERS-CoV, and open and closed forms of SARS-CoV-2 have, respectively, 9627, 10425, 8525, and 9202 active torsional degrees of freedom; however, AutoDock Vina could consider only 32 of them; thus, rigid docking was followed in our study.

### 2.5. Visualization

The protein-ligand interactions were analyzed and visualized using PyMOL 0.99rc6 [32] to study the 3-dimensional and surface annotation of atomic interactions between the receptor and ligand. Polar contacts between the molecules of ligand and receptor were determined at a distance of 5 Å for each binding mode. Inter atomic distances between residues having polar interactions were calculated for each binding mode.

### 2.6. Molecular Dynamic Analysis

Using the ligand-binding orientations calculated by molecular docking simulations, atomic simulations were performed for an NPT ensemble using the VMD 1.9.4 [33] and NAMD 2.14 [34, 35] packages with CHARMM36 [36] force field. Ligand docking poses obtained from AutoDock Vina were aligned with the original ligand molecule using the “align” command in PyMOL. Then, the PSF files of the aligned ligand molecule and receptor were created and merged into a single PSF file using VMD. All structures were solvated in water boxes having a minimum 15 Å cushion of water in each direction. In order to neutralize the solvation, ions were also added to the solvation, and NaCl concentrations were set to 0.15 mol/Lt to realize a typical biological environment. The sizes of the solvated systems in the closed and open states were about 360,000 atoms. Next, the MD simulations were completed using NAMD where the integration time step was 1 fs, periodic boundary conditions were applied, the Langevin dynamics was used to control the temperature and pressure of the ionized solvate, and all atoms were coupled to the heat bath. Equilibration of all systems was achieved in two steps. Firstly, a total of 10,000 steps of energy minimization were completed. Secondly, the minimized complex was subjected to MD simulations by setting the ensemble parameter to NPT (isothermal–isobaric ensemble, number of particles (*N*), pressure (*P*), and temperature (*T*)) at 310 K temperature and 1-bar pressure, and the total simulation run time was up to 50,010,000 steps (i.e., about 50 nanoseconds) in all MD simulations. Three separate MD simulations were run to warrant the stability of molecular interactions with the critical epitopes of Lys63 and Asp65 that have run times of 8, 20, and 50 nanoseconds. The convergence of the simulations was confirmed by monitoring the RMSD, temperature, the Van der Waals energy, the number of hydrogen bonds, potential energy, and binding energy variations throughout the simulations (e.g., see Suppl. Figures 1 and 2).

## 3. Results and Discussion

### 3.1. Interaction of Open-State SARS-CoV-2 or SARS-CoV Spike Proteins with CD147 Reveals Key Residues in CD147

Cryoelectron micrographs showed three populations of spike protein forms: a closed form (34%), an intermediate form (39%), and an open form (27%) [37]. Closed conformation of the spike protein structure resembles the uncleaved form [38]. In the closed conformation, the receptor-binding domain (RBD) surface, which is interacting with the hACE2 receptor, is buried inside the trimer and is not accessible for receptor binding. However, in the open conformation, the ACE2-interacting surface becomes fully exposed, therefore accessible for receptor binding [39].

Host cell infection by SARS-CoV-2 has been proposed to be via CD147, as an alternative novel route, and the localization of the interaction between the S protein and CD147 was shown by electron microscopy [40]. CD147 expression is known to increase in activated T cells as compared to resting T cells [17]. Because CD147 is implicated in the production of matrix metalloproteinase 9 (MMP9) that results in tissue remodeling, proinflammatory cytokine production [41], we aimed to identify the specific regions of CD147 that could potentially be used by SARS-CoV-2 as a binding interface with its spike protein.

**Figure 1:**
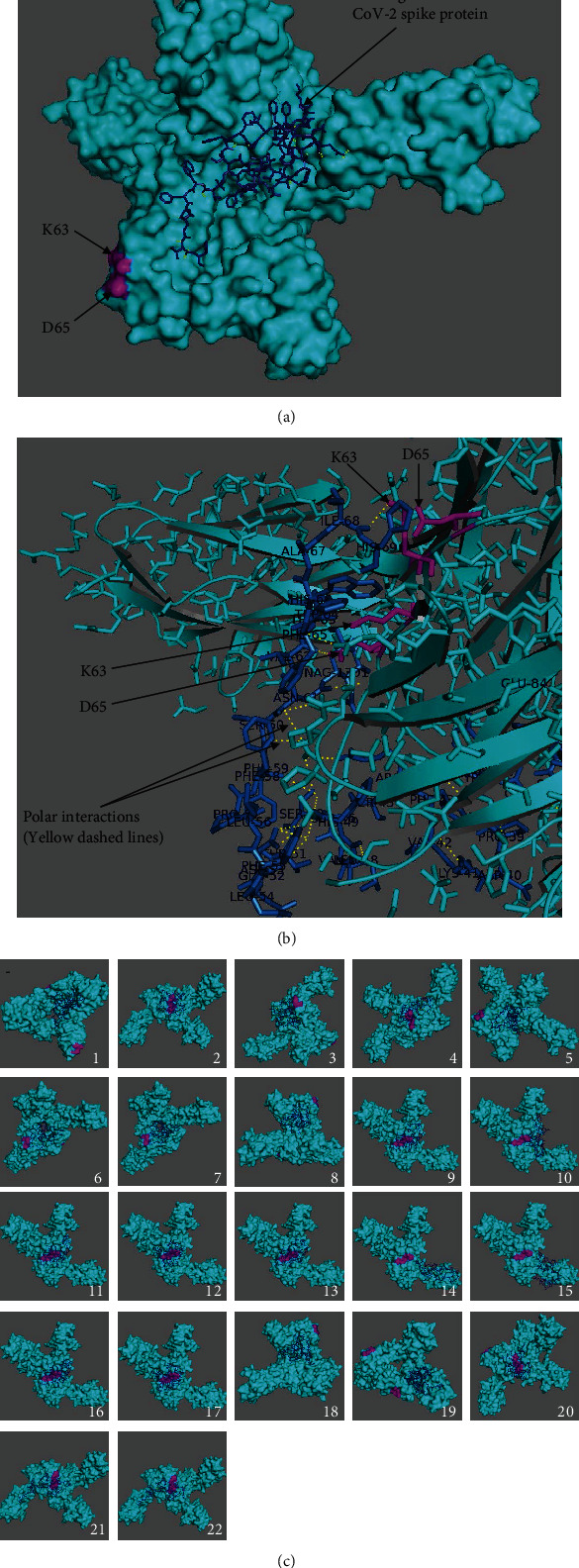
Open-state SARS-CoV-2 (PDB ID: 6VYB) docked in the receptor CD147 (PDB ID: 3B5H) with crystallographic water molecules: (a) the best binding mode in the receptor, (b) in the mode number 6 along with side chains as lines, and (c) the twenty-two best binding modes showing amino acid residues involved in the interactions at 5 Å distance (receptor in cyan, ligand as dark blue sticks, Lys63 and Asp65 residues in magenta, and interactions as yellow dashes).

Suggested by data on the relationship between CD147 and RA [17], docking simulations of the spike protein binding to CD147 led to the specific investigation of epitopes Lys63 (K63) and Asp65 (D65) and their involvement in the interaction with open and closed states of SARS-CoV-2 spike protein PDB ID: 6VYB. Interestingly, polar interactions based on the free binding energies (Table 1, Suppl. Tables 1-2) and docking snapshots (Figure 1) calculated by AutoDock Vina simulations suggested that these specific residues may be the key players in the interaction between the spike protein and CD147 PDB ID: 3B5H.

Data from the binding analysis with crystallographic water molecules is presented in Figure 1, Table 1, and Suppl. Table 1. Results of the twenty-two best binding modes between CD147 (PDB ID: 3B5H) as a receptor and open-state SARS-CoV-2 (PDB ID: 6VYB) as a ligand are presented, and free binding energies and RMSD values of binding modes were obtained using AutoDock Vina with a grid box of (150 Å × 120 Å × 120 Å) centered at 12.339 Å, -33.216 Å, and -11.505 Å with the exhaustiveness value of 12 (Table 1). The best binding mode between open-state SARS-CoV-2 docked in the receptor CD147 (Figure 1(a)); the interactions in the best binding mode at 5 Å distance (Figure 1(b)) and the twenty-two best binding modes of the CD147 receptor (Figure 1(c)) are shown (Figure 1). The Lys63 and Asp65 residues are shown in magenta to highlight the interactions with these residues. The 6th, 19th, 21st, and 22nd modes had interactions with Lys63 and Asp65 residues of Chains B and C in the receptor CD147 (Suppl. Table 1). The distances less than 5 Å among the interacting amino acids of open-state SAR-CoV-2 and residues of Lys63 and Asp65 are listed (Suppl. Table 1). Particularly, the 6th and 21st binding modes showed polar interactions simultaneously with Lys63 and Asp65 with high affinity.

Next, results of the twenty best binding modes between CD147 without crystallographic water molecules and open-state SARS-CoV-2 were analyzed (Suppl. Table 2). It is noteworthy that the receptor CD147 in the absence of crystallographic water molecules had no polar interaction with Lys63 and Asp65. Based on the comparison of free binding energies of binding modes listed in Table 1 and Suppl. Table 2, removing the crystallographic water molecules in the receptor CD147 PDB model resulted in lower binding energy values. This is concomitant with the notion that the inclusion of crystallographic water molecules in the binding site of numerous protein structures markedly improved the efficacy and accuracy of docking searches [31]. Subsequently, docking results of the CD147 with crystallographic water molecules were reliable and predict that there were multiple polar interactions among the residues of open-state SARS-CoV-2 and residues Lys63 and Asp65 of the CD147 receptor. Furthermore, we included the SARS-CoV and MERS-CoV in this study to compare their binding residues in CD147 with SARS-CoV-2. Although SARS-CoV and SARS-CoV-2 had overlapping binding residues in CD147, MERS-CoV did not bind to CD147. Interestingly, SARS-CoV did not bind to the critical epitopes Lys63 and Asp65 (Table 2). Binding residues including common residues and Lys63 and Asp65 in CD147 for SARS-CoV-2 and SARS-CoV are, respectively, shown in Tables 1 and 2. Comparing the binding residues listed in Tables 1 and 2, we showed that interaction between SARS-CoV and CD147 shared 46 common residues with that of SARS-CoV-2 and CD147 throughout the first twenty binding modes. While we identified five residues unique to SARS-CoV, the number of residues specific to SARS-CoV-2 was 44 including Lys63 and Asp65 across the first twenty binding modes. In brief, our results suggested a new aspect for the severity of SARS-CoV-2 infections which was not observed during SARS-CoV and MERS-CoV infections.

Next, we completed the MD analyses using the binding modes predicted by AutoDock Vina. The open-state SARS-CoV-2 ligand molecule was aligned with the binding mode number 6 listed in Suppl. Table 1 using the PyMOL software. The binding mode number 6 in Suppl. Table 1 was selected since it had the highest affinity among the other binding modes. Then, we evaluated the equilibration of the system in two phases, i.e., an NVT ensemble (constant number of particles, volume, and temperature) followed by an NPT ensemble (constant number of particles, pressure, and temperature). Suppl. Figure 1 presents the RMSD, temperature, the Van der Waals Energy, number of hydrogen bonds, potential energy, and binding energy variations during 50 nanoseconds simulation. By examining the six plots in Suppl. Figure 1, it is concluded that the simulations converged. Each frame in Suppl. Figures corresponds to 100,000 femtoseconds, and the symbol TS denotes one femtosecond. In order to validate the stability of polar interactions with the residues Lys63 and Asp65, they were investigated at intermittent time steps, and we observed that there were robust polar interactions with the receptor residues Lys63 and Asp65 throughout the simulations. To this end, the three independent simulations were run for 8, 20, and 50 nanoseconds whose results are summarized in Suppl. Table 3. The ligand molecules interacting with the residues Lys63 and Asp65 may change in time but there were always multiple strong interactions with both Lys63 and Asp65. Suppl. Figure 1d implies that the number of polar interactions was stabilized and the total number of polar interactions was in a stable state. The binding energy variation given in Suppl. Figure 1f also confirmed the stability of interactions. Converged potential energy and the Van der Waals energy plots shown, respectively, in Suppl. Figures 1c and 1e indicated the establishment of stable interatomic interactions. Recall that there was an energy minimization stage in the first 10,000 femtoseconds and then the NPT stage started in simulations. These stages could be detected in the plots. Polar interactions with Lys63 and Asp65 were not interrupted in this stabilized simulation stage for the three independent simulations run for 8, 20, and 50 nanoseconds.

**Figure 2:**
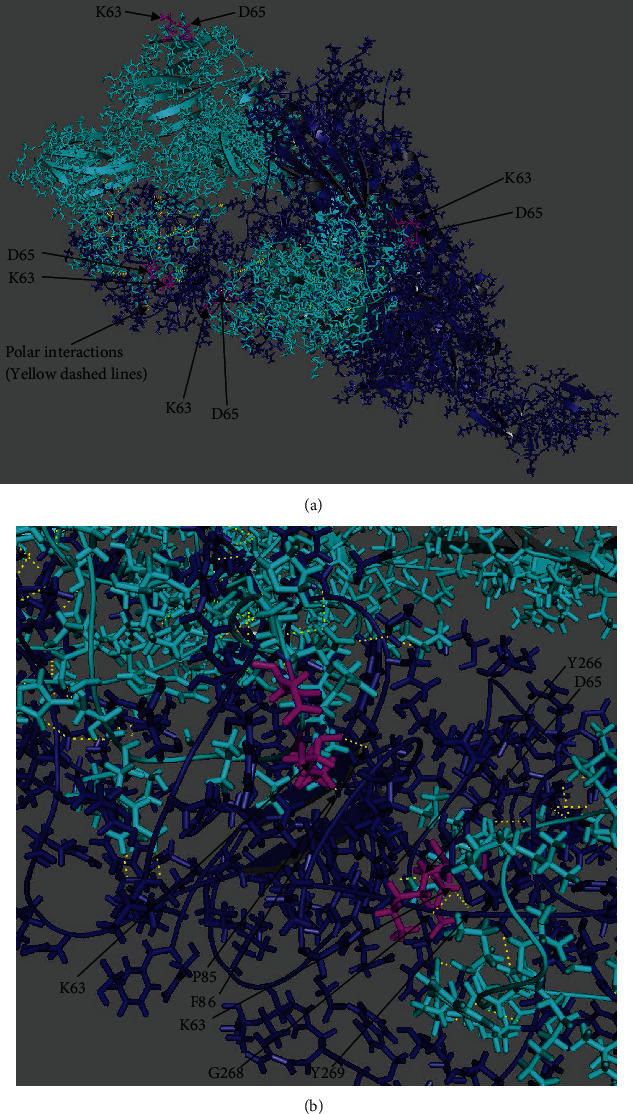
The last frame of the molecular dynamic simulation for the open-state SARS-CoV-2 (PDB ID: 6VYB) docked in the receptor CD147 (PDB ID: 3B5H) at the end of 50 nanoseconds (a) using the initial pose of binding mode 6 found by AutoDock Vina (wide view) and (b) using the initial pose of binding mode 6 found by AutoDock Vina (local view) (receptor in cyan, ligand as dark blue sticks, Lys63 and Asp65 residues in magenta, and interactions as yellow dashes). Note that all interactions in Suppl. Table 3 could not be shown due to the 2-dimensional view.

The polar interaction pairs at 5 Å distance at the end of simulation for 50 nanoseconds were Asp65-Tyr266 (3.11 Å), Lys63-Asn81 (2.17 Å), Lys63-Leu84 (1.99 Å, 4.37 Å), Lys63-Pro85 (3.00 Å), Lys63-Phe86 (2.06 Å), Lys63-Phe238 (4.91 Å), Lys63-Val267 (4.27 Å), Lys63-Gly268 (3.25 Å), and Lys63-Tyr269 (2.46 Å). The polar interaction pairs at 3 Å distance at the end of simulation for 50 nanoseconds were Lys63-Phe86 (2.06 Å), Lys63-Leu84 (1.99 Å), and Lys63-Tyr269 (2.46 Å). In brief, although the ligand residues interacting with the receptor residues Lys63 and Asp65 might change during the simulations, the bindings consistently involved the receptor residues Lys63 and Asp65 throughout the simulations. The polar interaction pairs of the residues Lys63 and Asp65 at 8, 20, and 50 nanoseconds are listed in Suppl. Table 3. The last frame of the MD simulation at the end of 50 nanoseconds for open-state SARS-CoV-2 (PDB ID: 6VYB) docked in the receptor CD147 (PDB ID: 3B5H) is shown in Figures 2(a) and 2(b). Results obtained from PyMOL clearly showed that the ligand had polar interactions with the receptor residues Lys63 and Asp65.

Since the hydrogen bonds were characterized as strong (2.2–2.5 Å), moderate (2.5–3.2 Å), and weak (3.2–4.0 Å) based on the distance between the donor and acceptor [14, 42–44], we concluded that the ligand had strong polar interactions with the receptor residues Lys63 and Asp65, given that the interatomic distances were less than 2 Å (Suppl. Table 3). Note that the other binding modes in Suppl. Table 1 were also examined in MD simulations as the initial poses, and it was observed that these binding modes also yielded polar interactions with the residues Lys63 and Asp65. The MD simulation results of the binding mode numbers 19, 21, and 22 in Suppl. Table 1 are not presented here due to limited space.

In a recent study, these same two crucial residues in CD147, i.e., Lys63 and Asp65, facilitated the production of hyperactivated memory T cells in rheumatoid arthritis [17]. Furthermore, Lys63 binding led to a higher level of hyperactive T cells when compared to Asp65 binding. In the case of the simultaneous binding with Lys63 and Asp65, the hyperactivation of T cells was even greater than that of any single binding case [17]. Altogether results from Guo et al. [17] and our study suggest that spike proteins may bind to Lys63 and Asp65 residues in CD147 and lead to an abnormal hyperactivation of T cells that in turn may potentially trigger T cell-mediated autoimmune responses. Finally, we screened the Delta (T19R, (V70F^∗^), T95I, G142D, E156-, F157-, R158G, (A222V^∗^), (W258L^∗^), (K417N^∗^), L452R, T478K, D614G, P681R, and D950N) and Omicron variants (A67V, del69-70, T95I, del142-144, Y145D, del211, L212I, ins214EPE, G339D, S371L, S373P, S375F, K417N, N440K, G446S, S477N, T478K, E484A, Q493R, G496S, Q498R, N501Y, Y505H, T547K, D614G, H655Y, N679K, P681H, N764K, D796Y, N856K, Q954H, N969K, and L981F) of SARS-CoV-2 open form and concluded that none of these mutations affected the polar interactions with critical residues Lys63 and Asp65 in CD147 (Suppl. Table 3).

### 3.2. CD147 and Closed-State SARS-CoV-2 Spike Protein Interactions

**Figure 3:**
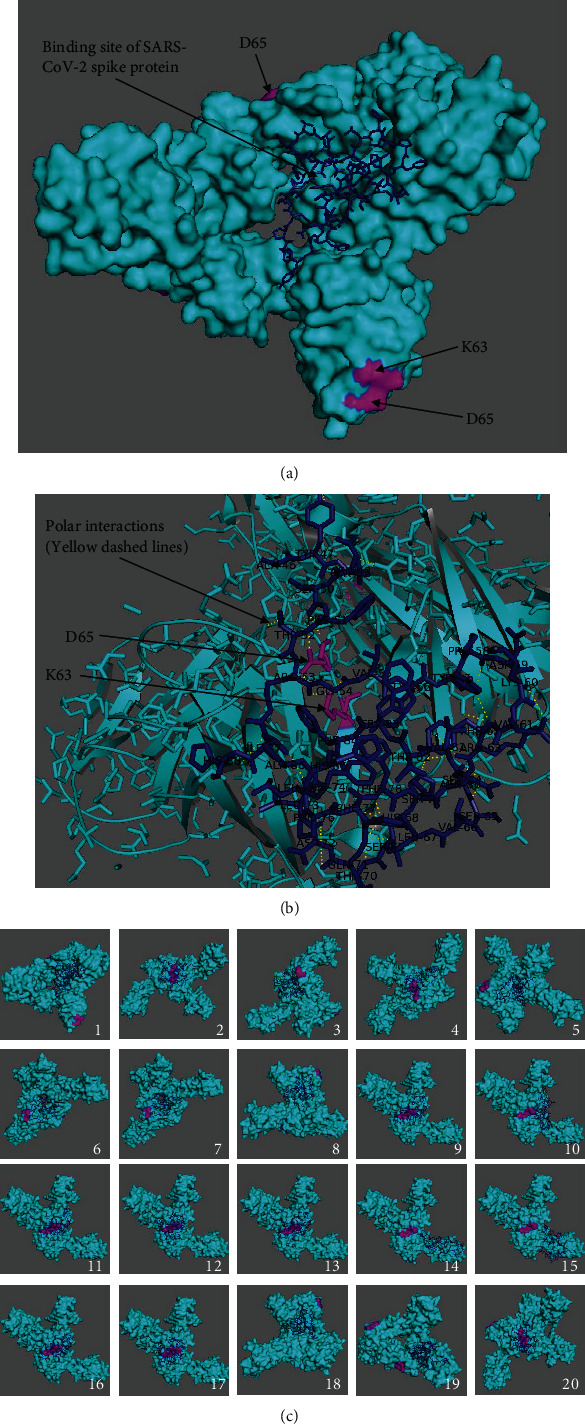
Closed-state SARS-CoV-2 (PDB ID: 6VXX) docked in the receptor CD147 (PDB ID: 3B5H) with crystallographic water molecules: (a) the best binding mode in the receptor, (b) in the mode number 2 along with side chains as lines, and (c) the best twenty binding modes showing amino acid residues involved in the interactions at 5 Å distance (receptor in cyan, ligand as dark blue sticks, Lys63 and Asp65 residues in magenta, and interactions as yellow dashes).

Results of the twenty best binding modes between CD147 as a receptor and closed-state SARS-CoV-2 (PDB ID: 6VXX) as a ligand are presented in Suppl. Tables 4 and 5. Free binding energies and RMSD values of docking experiments were obtained with AutoDock Vina using the same parameters as in Section 3.1. The best binding mode between closed-state SARS-CoV-2 docked in the receptor CD147 is presented in Figure 3(a); the interactions in the best binding mode at 5 Å distance are presented in Figure 3(b); and the twenty best binding modes with amino acid residues involved in the interactions at 5 Å distance are determined and presented in Figure 3(c).

Lys63 and Asp65 in CD147 are shown in magenta to highlight the docking with these residues (Figure 3). The 2nd, 4th, 9th, 10th,11th, 12th, 13th, 16th,17th, and 20th modes in Figure 3 had polar interactions with Lys63 and Asp65 residues in the receptor CD147 (Suppl. Table 5) where the distances among the interacting amino acids of the ligand SARS-CoV-2 and residues of Lys63 and Asp65 in CD147 were less than 5 Å. Computed energies and RMSD values of Lys63 and Asp65 interactions suggested that there is a possibility of hyperactivation of T cells by CD147 despite the fact that the SARS-CoV-2 ligand was in a closed state (Suppl. Table 5). Of all the binding modes exerting interactions with the epitopes, the 4th mode was the only mode without simultaneous polar interactions with Lys63 and Asp65. It is noteworthy that the free binding energies of closed-state spike protein and CD147 were lower than those of open-state spike protein, indicating that closed spike protein has a lower affinity than the open-state form.

As shown in Suppl. Tables 1 and 5, free binding energies of the best binding modes of open-state SARS-CoV-2 were relatively higher than those of the closed-state SARS-CoV-2. This clearly indicates a higher binding affinity for open-state SARS-CoV-2. Interestingly, the number of binding modes for closed-state SARS-CoV-2 in binding with Lys63 and Asp65 in CD147 is larger (10 out of 20) than those of open-state SARS-CoV-2 (4 out of 22).

Next, docking simulations were repeated by considering the receptor CD147 without crystallographic water molecules (Suppl. Tables 6 and 7). The free binding energies of binding modes for the CD147 without crystallographic water molecules are much lower than those of the CD147 with crystallographic water molecules. There was only one binding mode (i.e., the 11th binding mode) in which the residuals both Lys63 and Asp65 had simultaneous polar interactions with SARS-CoV-2 of which details are given in Suppl. Table 7. In all binding modes, except the 18th, docking of molecules yielded polar interactions around the root of chains of CD147. Lastly, we screened the Delta and Omicron variants of SARS-CoV-2 closed form and verified that these mutations did not affect the polar interactions with critical residues Lys63 and Asp65 in CD147 (Suppl. Table 8).

Similar to the open-state SARS-CoV-2 ligand, MD analyses were completed using the binding modes predicted by AutoDock Vina. A closed-state SARS-CoV-2 ligand molecule was aligned with the binding mode number 2 listed in Suppl. Table 5 using the PyMOL software. The binding mode number 2 was selected because it had the highest affinity among the binding modes having polar interactions with the residues Lys63 and Asp65. Following, the equilibration of the system was evaluated in two phases (i.e., firstly an NVT ensemble, then an NPT ensemble). Suppl. Figure 2 demonstrates the RMSD, temperature, the Van der Waals energy, number of hydrogen bonds, potential energy, and binding energy variations during 50 nanoseconds simulation. By examining the six plots in Suppl. Figure 2, it is concluded that the simulations converged. In order to examine the stability of polar interactions with the receptor residues Lys63 and Asp65, they were traced at intermittent time steps, and we observed that there were robust polar interactions with the receptor residues Lys63 and Asp65 throughout the simulations. To this end, the three independent simulations were run for 8, 20, and 50 nanoseconds whose results are summarized in Suppl. Table 8. Similar to open-state spike protein, there were always multiple interactions with both Lys63 and Asp65. Suppl. Figure 2d implies that the number of polar interactions was stabilized. Recall that there was an energy minimization stage in the first 10,000 femtoseconds, and then the NPT stage started in simulations. These stages could be detected in the plots. The converged potential energy, Van der Waals energy, and binding energy plots shown, respectively, in Suppl. Figures 2c, 2e, and 2f indicated the establishment of stable interatomic interactions in the NPT stage. Polar interactions with Lys63 and Asp65 were not interrupted in this stabilized simulation stage for the three independent simulations run for 8, 20, and 50 nanoseconds.

**Figure 4:**
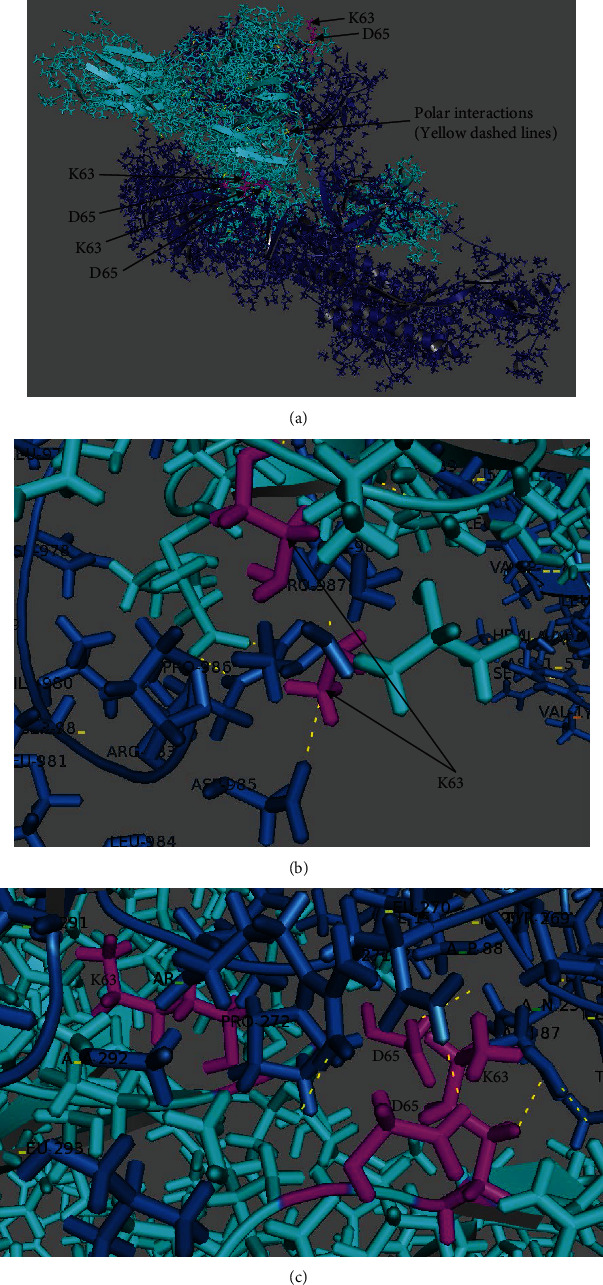
The last frame of the MD simulation for the closed-state SARS-CoV-2 (PDB ID: 6VXX) docked in the receptor CD147 (PDB ID: 3B5H)) at the end of 50 nanoseconds (a) using the initial pose of binding mode 2 found by AutoDock Vina (wide view) and (b, c) using the initial pose of binding mode 2 found by AutoDock Vina (local views) (receptor in cyan, ligand as dark blue sticks, Lys63 and Asp65 residues in magenta, and interactions as yellow dashes). Note that all interactions in Suppl. Table 3 could not be shown due to the 2-dimensional view.

The polar interaction pairs at distance 5 Å at the end of 50 nanoseconds were Asp65-Gln271 (3.12 Å, 3.25 Å), Asp65-Asp88 (3.17 Å), Lys63-Asp985 (2.63 Å), Lys63-Pro986 (4.91 Å), and Lys63-Pro987 (2.03 Å). The polar interaction pair of the residues Lys63 and Asp65 at distance 3 Å at the end of 50 nanoseconds was Lys63-Pro987 (2.03 Å). Briefly, the ligand had strong polar interaction with the receptor residues Lys63 and Asp65 due to the Lys63-Pro987 interaction with a distance of 2.03 Å. The polar interaction pairs of the residues Lys63 and Asp65 at 8, 20, and 50 nanoseconds are listed in Suppl. Table 8. It is observed that the number of polar interaction pairs of the residues Lys63 and Asp65 reduced in time for the closed-state SARS-CoV-2 (Suppl. Table 8) while it was stable for the open-state SARS-CoV-2 (Suppl. Table 3). Moreover, the number of interactions between closed-state SARS-CoV-2 with the residues Lys63 and Asp65 (Suppl. Table 3) is lower than that of open-state SARS-CoV-2 (Suppl. Table 8). By comparing between the interacting residues of the ligand and receptor given in Suppl. Tables 3 and 8, it was concluded that the binding residues of open-state SARS-CoV-2 and closed-state SARS-CoV-2 were completely different, and there was no overlap among these binding residues. Note that other binding modes in Suppl. Table 5 were also used in MD simulations as the initial poses, and it was observed that these binding modes also yielded polar interactions with the residues Lys63 and Asp65. The results of the other binding modes are not shown here due to limited space. The last frame of the MD simulation at the end of 50 nanoseconds for closed-state SARS-CoV-2 (PDB ID: 6VXX) docked in the receptor CD147 (PDB ID: 3B5H) is shown in Figures 4(a)–4(c) that were obtained from PyMOL.

### 3.3. Interaction of Retinal Specific CD147 with SARS-CoV-2 Spike Protein in Open and Closed States Did Not Involve Key Residues

**Figure 5:**
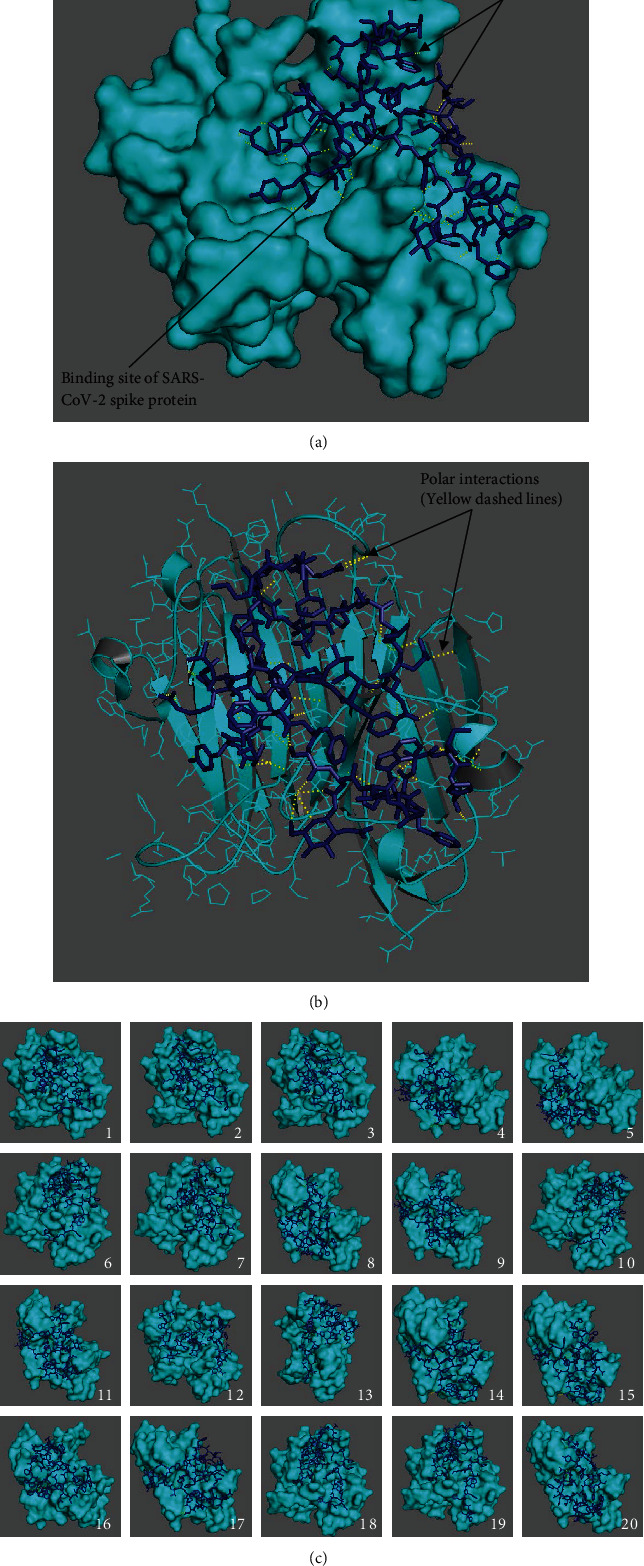
Open-state SARS-CoV-2 (PDB ID: 6VYB) docked in the retinal CD147 receptor (PDB ID: 3QR2) with crystallographic water molecules: (a) the best binding mode in the receptor, (b) the best binding mode in the receptor along with side chains as lines, and (c) the best twenty binding modes showing amino acid residues involved in the interactions at 5 Å distance (receptor in cyan, ligand as dark blue sticks, and interactions as yellow dashes).

**Figure 6:**
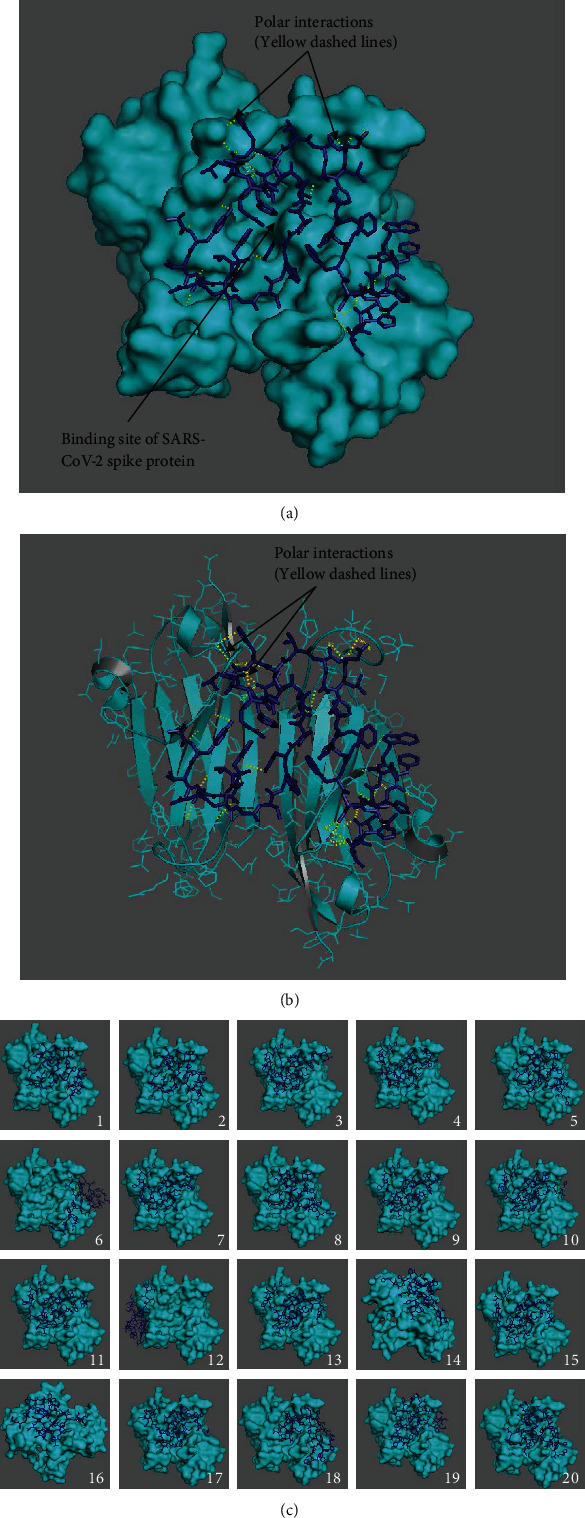
Closed-state SARS-CoV-2 (PDB ID: 6VYB) docked in the retinal CD147 receptor (PDB ID: 3QR2) with crystallographic water molecules: (a) the best binding mode in the receptor, (b) the best binding mode in the receptor along with side chains as lines, and (c) the best twenty binding modes showing amino acid residues involved in the interactions at 5 Å distance (receptor in cyan, ligand as dark blue sticks, and interactions as yellow dashes).

Then, we investigated the retinal specific CD147 about which we know little [12]. Although CD147 Ig1-Ig2 is ubiquitously expressed in most tissues, the CD147 Ig0 domain is retinal specific. To compare the retinal specific CD147 Ig0 with ubiquitous CD147, first, we analyzed the interaction of retinal specific wild-type CD147 Ig0 (PDB: 3QR2) and open-state spike protein and, secondly, retinal CD147 Ig0 and closed-state spike protein. Even though residues Lys63 and Asp65 are missing in retinal CD147, to complete all the comparisons of interactions, we determined the free binding energies from the spike proteins both at open and closed states and retinal CD147 in the same manner as the ubiquitously expressed CD147. Our results indicated that retinal specific CD147 residues had polar interactions with the residues of both open and closed SARS-CoV-2 spike proteins listed in Suppl. Tables 9-12 and Figures 5–6.

Results of twenty best binding modes between CD147 with crystallographic water molecules as a receptor and open-state SARS-CoV-2 as a ligand are presented (Suppl. Table 9). Free binding energies and RMSD values of docking experiments were obtained with AutoDock Vina using a grid box of (150 Å × 120 Å × 120 Å) centered at 14.512 Å, -17.972 Å, and -9.335 Å with the exhaustiveness value of 12 (Suppl. Table 9-12).  Figure 5(a) presents the best binding mode; Figure 5(b) shows the interactions in the best binding mode at 5 Å distance; and Figure 5(c) shows the twenty best binding modes between open-state SARS-CoV-2 docked in retinal specific CD147 with crystallographic water molecules.

The binding analyses were repeated for the receptor without crystallographic water molecules whose results were presented in Suppl. Table 10. By comparing Suppl. Tables 9 and 10, removing the crystallographic water molecules resulted in lower free binding energies. Moreover, the free binding energies of retinal CD147 were lower than those of ubiquitous CD147. For example, the highest twenty free binding energies for spike protein open conformation were in the range of -5.6 to -4.3 kcal/mol for ubiquitous CD147, whereas they were in the range of -4.9 to -4.0 kcal/mol for retinal CD147.

The binding modes of the case without crystallographic water molecules were in the vicinity of the best binding mode while the binding modes of the case with crystallographic water molecules showed different binding sites. On one hand, these sites were close to the locations of the crystallographic water molecules, indicating that crystallographic water molecules attracted the residues of the ligand towards themselves and caused polar interactions between the residues of the ligand and residues of the receptor. On the other hand, the docking sites without crystallographic water molecules always included the region in between the chains A and B of the receptor while the docking sites with crystallographic water molecules were all around the receptor side chains.

Subsequently, docking simulations between retinal specific CD147 and closed-state SARS-CoV-2 spike protein were completed both in the presence and absence of crystallographic water molecules. The computational parameters in these docking trials were identical to those of the open-state SARS-CoV-2 ligand. Suppl. Tables 11 and 12 depict the corresponding free binding energies and RMSD values of the twenty best binding modes for the receptor with and without crystallographic water molecules, respectively. The binding sites and polar interactions of the twenty best binding modes in the presence of crystallographic water are given in Figure 6. All binding modes were in between the chains A and B of the receptor except for the binding mode numbers 6 and 12. Besides, the corresponding RMSD values showed that docking took place in the vicinity of the best binding mode except for the 6th, 12th, 14th, and 16th binding modes. In comparison to open-state SARS-CoV-2, closed-state SARS-CoV-2 had relatively lower free binding energies. On the other hand, docking sites of the retinal specific CD147 without crystallographic water molecules included the region in between the chains A and B of the receptor for both states. In parallel, omitting the crystallographic water molecules caused relatively lower free binding energies for both open and closed states.

In the open conformation, spike protein's free binding energies were found in the range of -5.8 to -3.9 kcal/mol for hACE2 surface interaction (data not shown), whereas they ranged from -5.6 to -4.3 kcal/mol for ubiquitous CD147 surface interaction (Table 1), suggesting a comparable affinity for both receptors. As the binding of the virus to the relevant receptor is a random collision event, the likelihood of spike protein binding to CD147 on the Lys63 and Asp65 would differ from one individual to the next, given that human populations are outbred and viral load would vary in each individual. Additionally, the single nucleotide polymorphism (SNP) in CD147 has been associated with the risk of cardiovascular diseases [45]. Hence, the combination of genetic polymorphism at the CD147 gene loci and SARS-CoV-2 fingerprint, having different immunologic imprinting in every individual, might explain the abnormal immune responses and long-term effects of COVID-19 in some individuals.

## 4. Conclusions

Since CD147 has roles in the production of matrix metalloproteinase 9 (MMP9) resulting in tissue remodeling, proinflammatory cytokine production, and autoimmune reactions [41, 46], we aimed to identify the key component of CD147 as a potential target for SARS-CoV-2 spike protein binding. We elucidated the binding of SARS-CoV-2 spike protein in both open- and closed-state forms with ubiquitously expressed CD147 utilizing molecular docking and MD simulations. Both MD and docking simulations suggested that Lys63 and Asp65 residues may be involved in the interaction of SARS-CoV-2 spike protein and CD147 based on free binding energies for open- and closed-state SARS-CoV-2. Open- and closed-state forms of SARS-CoV-2 spike protein created polar interactions with Lys63 and Asp65 on ubiquitous CD147. Interestingly, despite its lower binding affinity, SARS-CoV was also bound to CD147 in docking simulations; however, this interaction did not include Lys63 and Asp65 residues. On the other hand, MERS-CoV did not appear to bind CD147 at all in our simulations. Furthermore, Delta and Omicron variants of SARS-CoV-2 did not affect the polar interactions with critical residues Lys63 and Asp65 in CD147. Both molecular docking and MD simulations confirmed that this interaction is highly possible to encounter, in particular for open-state SARS-CoV-2. CD147 is known to be involved in abnormal immune responses seen in autoimmune diseases such as RA and may have similar relevance in the pathology of SARS-CoV-2. Known to be associated with the hyperactivation of memory T cells, CD147 might be causing abnormal activation of memory T cells (autoreactive or hyperreactive) in some COVID-19 patients. Most importantly, one recent study reported the presence of functionally diverse autoantibodies in COVID-19 patients, supporting our conclusions [46]. This study reveals alternative pathways for severe outcomes of SARS-CoV-2 infection and proposes novel targets for designing more efficient antivirals for the treatment of COVID-19 as well as other viral diseases, in particular, targeting at prevention of T cell overactivation.

## Figures and Tables

**Table 1:** Twenty best docking scores (lowest binding energy) in docking experiments between CD147 receptor with crystallographic water molecules and open-state SARS-CoV-2 ligand.

Mode	Free binding energy (kcal/mol)	Distance from RMSD lower bound	Distance from RMSD upper bound	Binding residues
1	-5.6	0	0	Chain B: V30, D32, Q100, H102, T135, and S190 Chain D: E84, S86, T96, N98, Q100, Q164, and S190
2	-5.4	25.503	37.483	Chain A: R54, E84, S86, E92, T96, R106, K127, E129, and R166 Chain B: S35 and S78
3	-5.2	25.333	37.519	Chain A: R54, E84, S86, E92, T96, R106, E114, N117, E120, K127, E129, and R166 Chain B: S35, D77, and S78 Chain C: N44
4	-5.2	14.004	30.153	Chain A: V30, T135, Q164, and K191 Chain B: T25, T29, V30, E31, D32, T40,and N44 Chain C: D32 and S35 Chain D: S78, H102, T135, S190, K191, and S193
5	-5.2	27.110	36.676	Chain A: G58, E84, E93, N98, K127, and R166 Chain B: S35, K36, D77, and D79 Chain C: E49
6	*-5.1*	*25.549*	*42.181*	Chain A: E84 and Q100 Chain B: S35 and *K63* Chain C: E31, S42, A47, T48, E49, T51, R54, *D65*, and K71
7	-5.1	1.539	2.311	Chain A: Y140 Chain B: T29, N98, H102, and S190 Chain D: E84, N98, Q100, E129, Q164, and S190
8	-5.1	5.276	18.087	Chain A: T135 and Y140 Chain B: V30 and H102 Chain D: G58, E84, T96, N98, Q100, V134, T135, Q164, R166, and S190
9	-5.1	24.041	40.490	Chain A: V30, E84, E92, N98, Q100, and H102 Chain B: L33, S35, K36, and K75 Chain C: E31, T40, N44, D45, S46, and Q70
10	-5.0	42.953	58.809	Chain A: E31, S42, N44, D45, and K71 Chain C: D136, Y140, A149, N152, E155, S162, T188, S190, and K191
11	-4.9	23.969	40.989	Chain A: V30, R54, G58, E84, E92, N98, H102, and S130 Chain B: K75 Chain C: E31, N44, D45, and S46
12	-4.9	42.937	58.925	Chain A: E30, T40, S42, N44, D45, and K71 Chain C: D136, Y140, A149, S162, T188, S190, K191, and G192
13	-4.9	5.442	17.814	Chain A: Y140 Chain B: V30, D32, I99, Q100, and H102 Chain D: K57, E84, T96, N98, Q100, K127, T135, Q164, and S190
14	-4.9	19.688	35.028	Chain A: T29, V30, E84, S86, E92, T96, and Q100 Chain B: D32, G34, S35, K36, D77, S78, D79, Q81, S193, and D194 Chain C: E31, D32, S46, Q70, K71, and E73
15	-4.9	28.147	42.202	Chain A: R54, G58, E84, S86, E92, T96, and Q100 Chain C: S42, N44, D45, S46, A47, V50, G69, Q70, and K71
16	-4.9	19.787	28.664	Chain B: S42, N44, D45, S46, P68, Q70, and K71 Chain C: D77 Chain D: D79, D80, W82, R106, S112, T143, D144, E146, D147, Q182, R184, N186, S193, D194, Q195, and T199
17	-4.9	3.816	5.797	Chain A: T135 Chain B: D32, N98, Q100, H102, T135, S189, and S190 Chain D: Q100, S130, Q164, R166, and S190
18	-4.9	24.508	35.673	Chain D: K108, V110, S112, S113, K141, I142, T143, D144, P180, Q182, I197, and T199
19	-4.8	37.421	53.789	Chain B: T51, R54, *K63*, and A66 Chain C: K57, D79, Q100, H102, G103, R106, K108, V110, K111, S113, E114, E129, and P132
20	-4.8	43.077	59.147	Chain A: E31, S42, L43, N44, D45, and K71 Chain D: T135, D136, Y140, A149, N152, G153, E155, V160, S162, T188, S189, S190, and K191
21	*-4.4*	*23.469*	*38.230*	Chain B: *K63*, *D65*, S86, and E92 Chain C: R53, K57, E84, Q100, G103, R106, and K108
22	-4.3	23.564	38.021	Chain B: T51, R54, *K63*, *D65*, and E92 Chain C: R54, K57, Q100, G103, R106, K108, and E129

**Table 2:** Twenty best docking scores (lowest binding energy) in docking experiments between CD147 receptor without crystallographic water molecules and SARS-CoV ligand.

Mode	Free binding energy (kcal/mol)	Distance from RMSD lower bound	Distance from RMSD upper bound	Binding residues
1	-4.5	0	0	B: S66, E92, N98, Q100, K127, E129, and Q164 D: D136 and S162
2	-4.4	21.582	30.732	B: T29, E31, N44, S46, Q70, and K71 D: D32, D144, D147, K191, D194, and Q195
3	-4.2	28.837	39.508	A: E92, N98, Q100, and S163 B: S35, D77, S78, D79, D80, and Y85 C: E49 and K71
4	-4.2	21.730	31.202	B: S42, N44, D45, P68, Q70, and K71 C: S35 and K36 D: R106, D144, D147, K191, and S193
5	-4.2	40.197	49.778	A: T29, V30, E31, and D32 C: S78, D79, Q81, H102, S112, R184, K191, S193, and Q195
6	-4.1	22.277	31.319	B: D45, Q70, and K71 C: S35 and D77 D: Q81, P104, D147, N186, T188, S193, D194, and Q195
7	-4.0	29.159	39.803	A: E92, N98, S163 B: S35, D77, S78, D79, D80, Q81, and Y85 C: N44, E49, and K71
8	-4.0	23.027	33.134	B: D45, Q70, and K71 C; K35 and D77 D: R184, N186, K191, S193, D194, and Q195
9	-4.0	21.828	31.443	B: S42, N44, D45, Q70, and K71 D: D32, D79, D144, D147, K191, S193, and D194
10	-4.0	2.064	3.303	B: R54, E84, E92, N98, K108, K127, and Q164 D: D136 and S162
11	-3.9	29.391	39.958	A: E84, E92, N98, and S163 B: S35, D77, S78, D80, Q81, and Y85
12	-3.9	23.314	33.347	B: S42, N44, D45, Q70, and K71 C: K35 D: Q81, D147, R184, K191, S193, and Q195
13	-3.9	21.692	31.419	A: K191 B: S42, N44, D45, Q70, and K71 C: S35 and K36 D: Y140, R184, N186, S193, D194, and Q195
14	-3.9	24.050	34.682	B: N44 and D45 D: K35, D77, S78, D79, D144, R184, S193, D194, and Q195
15	-3.8	22.630	31.680	B: Q70 D: Y140, R184, N186, S193, and Q195
16	-3.8	11.801	25.903	A: D136 and Q164 B: T25, V30, D32, Q100, H102, and K191 D: E84, Q100, H102, D136, and W137
17	-3.8	21.690	31.701	B: N44, Q70, and K71 C: S78 D: Q81, D144, D147, R184, K191, S193, D194, and Q195
18	-3.8	46.516	58.259	C: S112, D144, D147, Q182, R184, N186, T188, S193, and D194
19	-3.8	30.143	44.282	B: N44 and D45 D: K35, D77, S78, D79, D80, W82, Y85, P104, and K108
20	-3.8	23.029	32.587	B: N44, D45, and Q70 D: R106, D144, D147, R184, N186, and S193

## Data Availability

The data used to support the findings of this study are included within the article.

## Abbreviations

COVID-19: Coronavirus disease
SARS-CoV: Severe acute respiratory syndrome
MERS: Middle East respiratory syndrome
TLR: Toll-like receptor
SLE: Systemic lupus erythematosus
Treg: Regulatory T cell
MMP: Matrix metalloproteinase
Ig: Immunoglobulin
RA: Rheumatoid arthritis
hACE2: Human angiotensin-converting enzyme 2
MD: Molecular docking
Asp: Aspartic acid
Lys: Lysine
IRF7: Interferon regulatory factor
IFN: Interferon
HIV: Human immunodeficiency virus
MD: Molecular dynamics
PDB: Protein data bank

## Supplementary Materials

Supplementary Materials Supplementary Table 1: binding modes having interactions between Lys63 and/or Asp65 residues of CD147 receptor with crystallographic water molecules and open-state SARS-CoV-2 ligand. Supplementary Table 2: twenty best docking scores (lowest binding energy) in docking experiments between CD147 receptor without crystallographic water molecules and open-state SARS-CoV-2 ligand. Supplementary Table 3: Delta and Omicron mutations in SARs-CoV-2 do not fall on the region where SARS-CoV-2 open form bind to Lys63 and Asp65 in CD147 (modes without the critical residues are not included in the table). Supplementary Table 4: twenty best docking scores (lowest binding energy) in docking experiments between CD147 receptor with crystallographic water molecules and closed-state SARS-CoV-2 ligand. Supplementary Table 5: binding modes having interactions between Lys63 and/or Asp65 residues of CD147 receptor with crystallographic water molecules and the closed-state SARS-CoV-2 ligand. Supplementary Table 6: twenty best docking scores (lowest binding energy) in docking experiments between CD147 receptor without crystallographic water molecules and closed-state SARS-CoV-2 ligand. Supplementary Table 7: binding modes having interactions between Lys63 and/or Asp65 residues of CD147 receptor without crystallographic water molecules and closed-state SARS-CoV-2 ligand. Supplementary Table 8: Delta and Omicron mutations in SARS-CoV-2 do not fall on the region where SARS-CoV-2 closed form binds to Lys63 and Asp65 in CD147 (modes without the critical residues are not included in the table). Supplementary Table 9: twenty best docking scores (lowest binding energy) in docking experiments between retinal specific CD147 receptor with crystallographic water molecules and open-state SARS-CoV-2 ligand. Supplementary Table 10: twenty best docking scores (lowest binding energy) in docking experiments between retinal specific CD147 receptor without crystallographic water molecules and open-state SARS-CoV-2 ligand. Supplementary Table 11: twenty best docking scores (lowest binding energy) in docking experiments between retinal specific CD147 receptor with crystallographic water molecules and closed-state SARS-CoV-2 ligand. Supplementary Table 12: twenty best docking scores (lowest binding energy) in docking experiments between retinal specific CD147 receptor without crystallographic water molecules and closed-state SARS-CoV-2 ligand. Supplementary Figure 1: convergence plots in molecular dynamics simulations using NAMD 1.9.4 for open-state SARS-CoV-2 (PDB ID: 6VYB) docked in the receptor CD147 (PDB ID: 3B5H) where the binding mode number 6 in Table 4 was used to align the ligand molecule as the initial configuration (a) RMSD, (b) temperature, (c) the Van der Waals energy, (d) number of hydrogen bonds, (e) potential energy, and (f) bond energy variations. Supplementary Figure 2: convergence plots in molecular dynamics simulations using NAMD 1.9.4 for closed-state SARS-CoV-2 (PDB ID: 6VXX) docked in the receptor CD147 (PDB ID: 3B5H) where the binding mode number 2 in Table 8 was used to align the ligand molecule as the initial configuration with restart options (a) RMSD, (b) temperature, (c) the Van der Waals energy, (d) number of hydrogen bonds, (e) potential energy, and (f) bond energy variations.
