# Supplementary material for: Computational Modeling of T Cell Hypersensitivity during Coronavirus Infections Leading to Autoimmunity and Lethality
Source: Comput Math Methods Med. 2022 Mar 22;2022:9444502. doi: 10.1155/2022/9444502 (PMC8948601; doi:10.1155/2022/9444502)
Supplement: Supplementary Materials — Supplementary Table 1: binding modes having interactions between Lys63 and/or Asp65 residues of CD147 receptor with crystallographic water molecules and open-state SARS-CoV-2 ligand. Supplementary Table 2: twenty best docking scores (lowest binding energy) in docking experiments between CD147 receptor without crystallographic water molecules and open-state SARS-CoV-2 ligand. Supplementary Table 3: Delta and Omicron mutations in SARs-CoV-2 do not fall on the region where SARS-CoV-2 open form bind to Lys63 and Asp65 in CD147 (modes without the critical residues are not included in the table). Supplementary Table 4: twenty best docking scores (lowest binding energy) in docking experiments between CD147 receptor with crystallographic water molecules and closed-state SARS-CoV-2 ligand. Supplementary Table 5: binding modes having interactions between Lys63 and/or Asp65 residues of CD147 receptor with crystallographic water molecules and the closed-state SARS-CoV-2 ligand. Supplementary Table 6: twenty best docking scores (lowest binding energy) in docking experiments between CD147 receptor without crystallographic water molecules and closed-state SARS-CoV-2 ligand. Supplementary Table 7: binding modes having interactions between Lys63 and/or Asp65 residues of CD147 receptor without crystallographic water molecules and closed-state SARS-CoV-2 ligand. Supplementary Table 8: Delta and Omicron mutations in SARS-CoV-2 do not fall on the region where SARS-CoV-2 closed form binds to Lys63 and Asp65 in CD147 (modes without the critical residues are not included in the table). Supplementary Table 9: twenty best docking scores (lowest binding energy) in docking experiments between retinal specific CD147 receptor with crystallographic water molecules and open-state SARS-CoV-2 ligand. Supplementary Table 10: twenty best docking scores (lowest binding energy) in docking experiments between retinal specific CD147 receptor without crystallographic water molecules and open-state SARS-CoV [file 9444502.f1.zip › Supplementary Figure 1_revised1.docx]

**Supplementary Figure 1.**

**A**


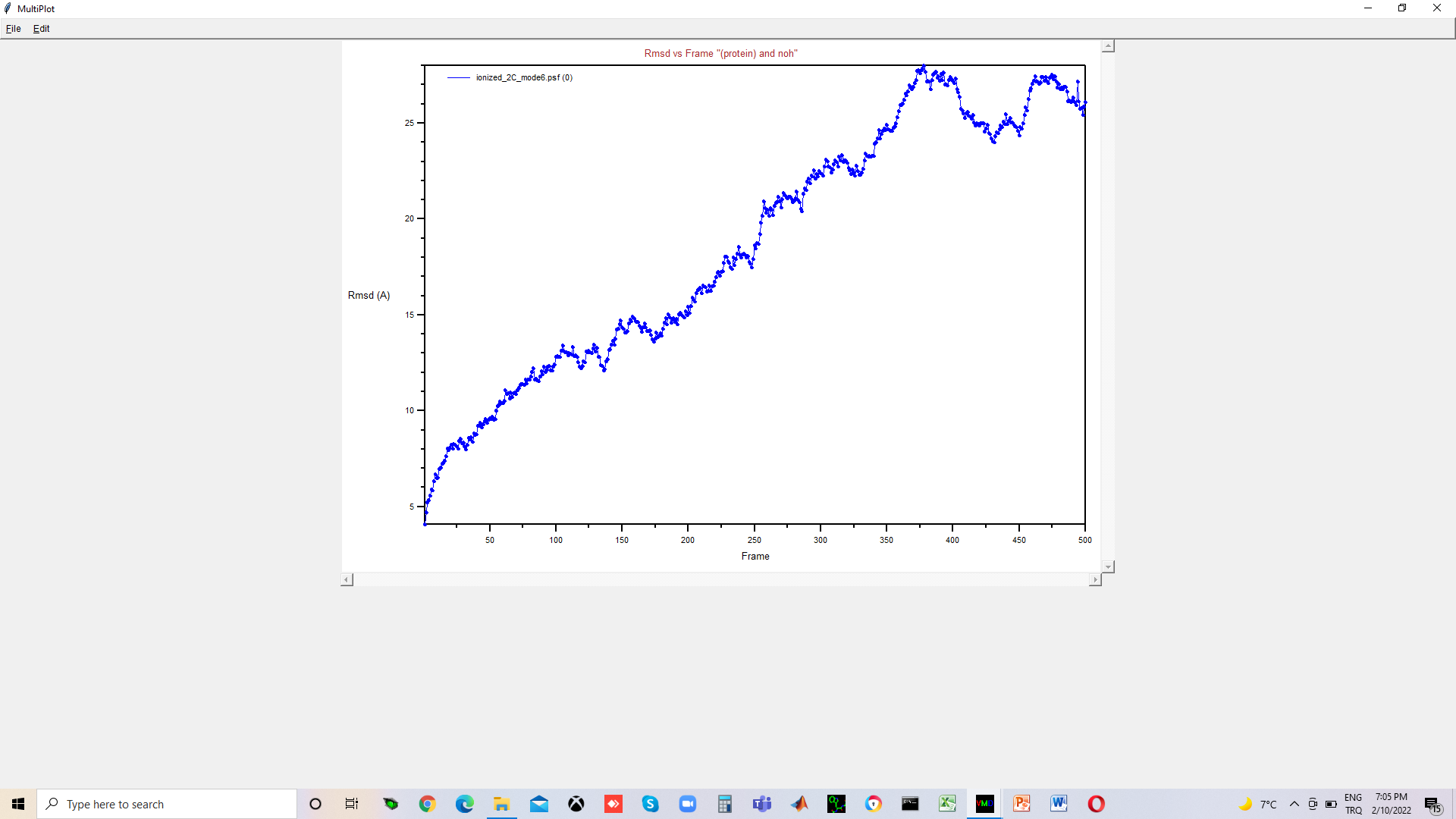


**B**


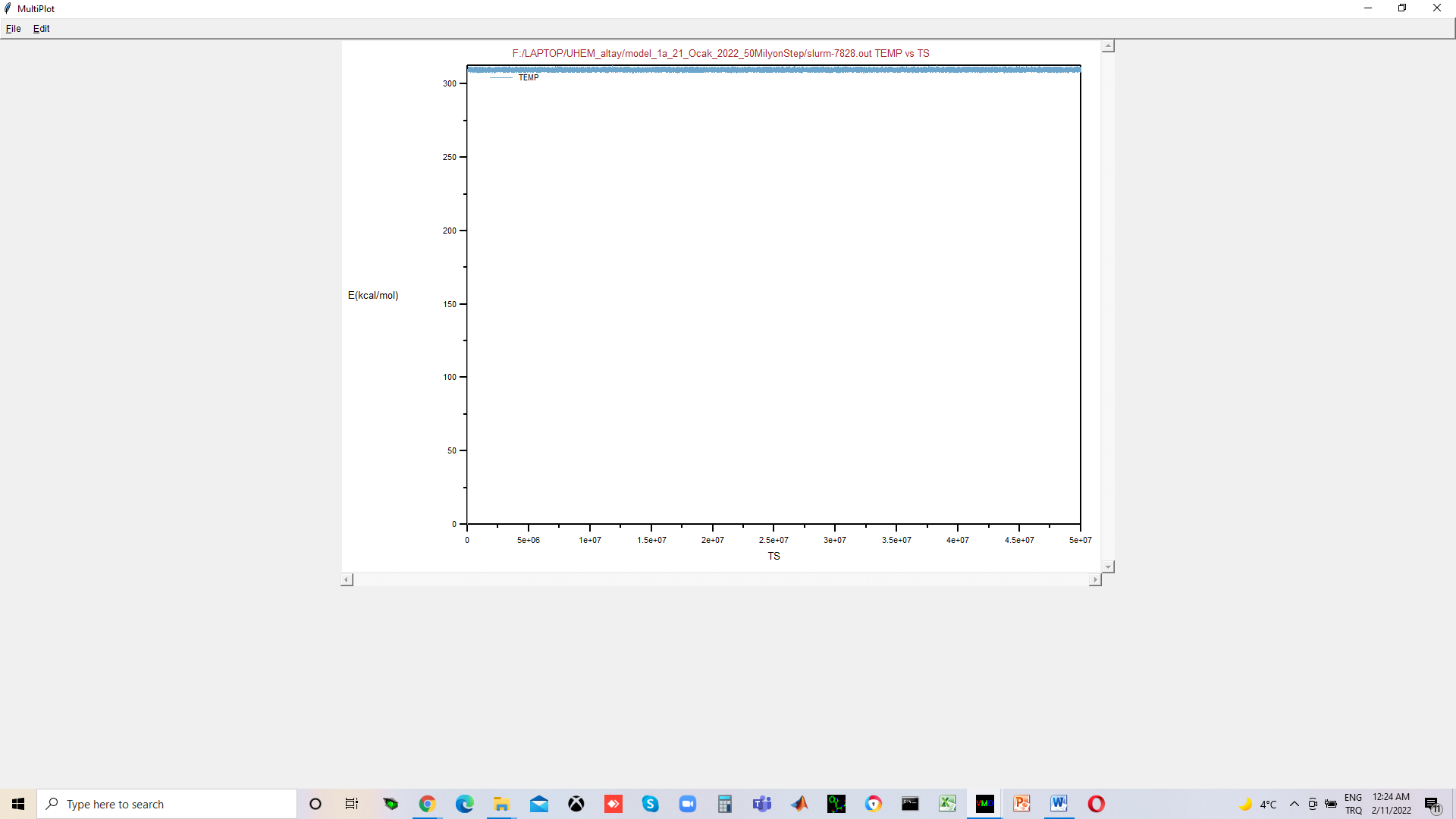


**C**

**D**


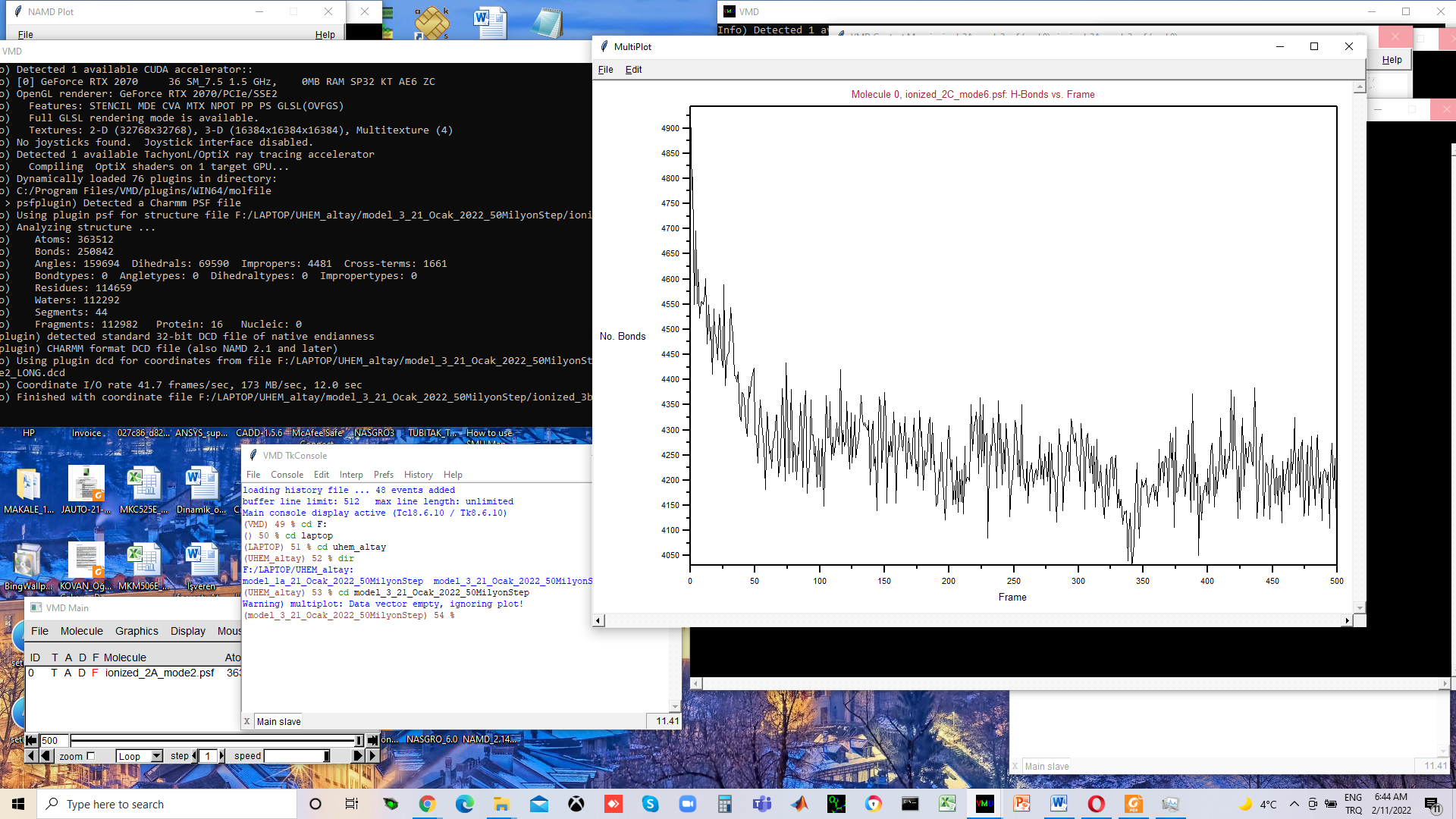


**E**

**F**

**Supplementary Figure 1. Convergence plots in molecular dynamic simulations using NAMD 1.9.4 for open state SARS-CoV-2 (PDB ID: 6VYB) docked in the receptor CD147 (PDB ID: 3B5H) where the binding mode number 6 in Table 4 was used to align the ligand molecule as the initial configuration (a) RMSD, (b) temperature, (c) Van der Waals energy, (d) number of hydrogen bounds, (e) potential energy and (f) bond energy variations.**
