# Supplementary material for: Computational Modeling of T Cell Hypersensitivity during Coronavirus Infections Leading to Autoimmunity and Lethality
Source: Comput Math Methods Med. 2022 Mar 22;2022:9444502. doi: 10.1155/2022/9444502 (PMC8948601; doi:10.1155/2022/9444502)
Supplement: Supplementary Materials — Supplementary Table 1: binding modes having interactions between Lys63 and/or Asp65 residues of CD147 receptor with crystallographic water molecules and open-state SARS-CoV-2 ligand. Supplementary Table 2: twenty best docking scores (lowest binding energy) in docking experiments between CD147 receptor without crystallographic water molecules and open-state SARS-CoV-2 ligand. Supplementary Table 3: Delta and Omicron mutations in SARs-CoV-2 do not fall on the region where SARS-CoV-2 open form bind to Lys63 and Asp65 in CD147 (modes without the critical residues are not included in the table). Supplementary Table 4: twenty best docking scores (lowest binding energy) in docking experiments between CD147 receptor with crystallographic water molecules and closed-state SARS-CoV-2 ligand. Supplementary Table 5: binding modes having interactions between Lys63 and/or Asp65 residues of CD147 receptor with crystallographic water molecules and the closed-state SARS-CoV-2 ligand. Supplementary Table 6: twenty best docking scores (lowest binding energy) in docking experiments between CD147 receptor without crystallographic water molecules and closed-state SARS-CoV-2 ligand. Supplementary Table 7: binding modes having interactions between Lys63 and/or Asp65 residues of CD147 receptor without crystallographic water molecules and closed-state SARS-CoV-2 ligand. Supplementary Table 8: Delta and Omicron mutations in SARS-CoV-2 do not fall on the region where SARS-CoV-2 closed form binds to Lys63 and Asp65 in CD147 (modes without the critical residues are not included in the table). Supplementary Table 9: twenty best docking scores (lowest binding energy) in docking experiments between retinal specific CD147 receptor with crystallographic water molecules and open-state SARS-CoV-2 ligand. Supplementary Table 10: twenty best docking scores (lowest binding energy) in docking experiments between retinal specific CD147 receptor without crystallographic water molecules and open-state SARS-CoV [file 9444502.f1.zip › Supplementary Tables_CMMM_CC_revised1.docx]

**Supplementary Data**

**SUPPLEMENTARY TABLES:**

**Supplementary Table 1: Binding modes having interactions between Lys63 and/or Asp65 residues of CD147 receptor with crystallographic water molecules and open state SARS-CoV-2 ligand.**

| **Mode** | **Free Binding Energy (kcal/mol)** | **Polar interaction distance (Å)** | **Interacting residues** | |
| --- | --- | --- | --- | --- |
|  |  |  | **CD147** | **SARS-CoV-2** |
| 6 | -5.1 | 3.54 | Asp65 in Chain C | V62 |
|  |  | 2.93 | Lys63 in Chain B | I68 |
| 19 | -4.8 | 3.16 | Lys63 in Chain B | I68 |
| 21 | -4.4 | 3.24 | Asp65 in Chain B | D45 |
|  |  | 3.23 | Asp65 in Chain B | D41 |
|  |  | 3.22 | Asp65 in Chain B | Q52 |
|  |  | 3.20 | Asp65 in Chain B | D53 |
|  |  | 3.08 | Lys63 in Chain B | W64 |
|  |  | 2.94 | Lys63 in Chain B | D53 |
| 22 | -4.3 | 3.39 | Lys63 in Chain B | Q52 |
|  |  | 2.98 | Lys63 in Chain B | W64 |
|  |  | 2.85 | Lys63 in Chain B | D53 |

**Supplementary Table 2: Twenty best docking scores (lowest binding energy) in docking experiments between CD147 receptor without crystallographic water molecules and open state SARS-CoV-2 ligand.**

| **Mode** | **Free Binding Energy (kcal/mol)** | **Distance from RMSD lower bound** | **Distance from RMSD upper bound** |
| --- | --- | --- | --- |
| 1 | -3.8 | 0 | 0 |
| 2 | -3.8 | 23.941 | 37.132 |
| 3 | -3.6 | 24.048 | 37.582 |
| 4 | -3.5 | 2.381 | 7.654 |
| 5 | -3.5 | 33.582 | 46.666 |
| 6 | -3.4 | 25.281 | 40.977 |
| 7 | -3.4 | 2.852 | 8.588 |
| 8 | -3.4 | 23.331 | 35.687 |
| 9 | -3.3 | 9.621 | 21.653 |
| 10 | -3.2 | 9.737 | 21.811 |
| 11 | -3.2 | 24.238 | 37.751 |
| 12 | -3.2 | 16.883 | 28.466 |
| 13 | -3.2 | 5.322 | 18.740 |
| 14 | -3.2 | 23.519 | 37.676 |
| 15 | -3.2 | 25.590 | 41.363 |
| 16 | -3.2 | 23.731 | 37.520 |
| 17 | -3.1 | 23.816 | 37.330 |
| 18 | -3.1 | 2.518 | 7.575 |
| 19 | -3.1 | 23.914 | 37.122 |
| 20 | -3.1 | 28.240 | 44.476 |

**Supplementary Table 3: Delta and Omicron mutations in SARS-CoV-2 do not fall on the region where SARS-CoV-2 open form bind to Lys63 and Asp65 in CD147, (Modes without the critical residues are not included in the table). Source for mutant variants:** [**https://www.cdc.gov/coronavirus/2019-ncov/variants/variant-info.html**](https://www.cdc.gov/coronavirus/2019-ncov/variants/variant-info.html)

| **Residues in SARS-CoV-2 having interactions** | | | **Mutations in SARS-CoV-2** |
| --- | --- | --- | --- |
| **Binding mode #** | | **Binding residues** | Delta and Omicron variants |
| **Docking mode # 6** | | .268/A/V62, I68 |  |
| **Docking mode # 19** | | .268/A/I68 |  |
| **Docking mode # 21** | | .228/A/D54; .229/A/Q52; .268/A/D53, W64 |  |
| **Docking mode # 22** | | .229/A/Q52; .268/A/D53, W64 |  |
| **MD results having polar interaction pairs at distance 5 Å** | **8 nanoseconds** | Asp65-Ser31, Asp65-Val62, Asp65-Arg237, Asp65-Gly268, Asp65-Tyr269, Lys63-Leu84, Lys63-Pro85, Lys63-Phe86, Lys63-Phe238, Lys63-Tyr265, Lys63-Val267 |  |
|  | **20 nanoseconds** | Asp65-Val90, Asp65-Tyr91, Asp65-Arg237, Asp65-Tyr265, Asp65-Gly268, Lys63-Phe86, Lys63-Gly268 |  |
|  | **50 nanoseconds** | Asp65-Tyr266 (3.11 Å), Lys63-Asn81 (2.17 Å), Lys63-Leu84 (1.99 Å, 4.37 Å), Lys63-Pro85 (3.00 Å), Lys63-Phe86 (2.06 Å), Lys63-Phe238 (4.91 Å), Lys63-Val267 (4.27 Å), Lys63-Gly268 (3.25 Å), Lys63-Tyr269 (2.46 Å) |  |
| **MD results having polar interaction pairs at distance 3 Å** | **8 nanoseconds** | Asp65-Ser31, Asp65-Gly268 |  |
|  | **20 nanoseconds** | Asp65-Arg237 |  |
|  | **50 nanoseconds** | Lys63-Phe86 (2.06 Å), Lys63-Leu84 (1.99 Å), Lys63-Tyr269 (2.46 Å) |  |

**Supplementary Table 4: Twenty best docking scores (lowest binding energy) in docking experiments between CD147 receptor with crystallographic water molecules and closed state SARS-CoV-2 ligand.**

| **Mode** | **Free Binding Energy (kcal/mol)** | **Distance from RMSD lower bound** | **Distance from RMSD upper bound** |
| --- | --- | --- | --- |
| 1 | -4.4 | 0 | 0 |
| 2 | -4.3 | 28.036 | 39.148 |
| 3 | -4.3 | 26.960 | 45.504 |
| 4 | -4.3 | 24.171 | 35.109 |
| 5 | -4.3 | 21.810 | 32.209 |
| 6 | -4.2 | 27.583 | 45.942 |
| 7 | -4.2 | 27.507 | 45.843 |
| 8 | -4.2 | 21.826 | 32.504 |
| 9 | -4.2 | 23.194 | 34.779 |
| 10 | -4.1 | 18.039 | 29.522 |
| 11 | -4.1 | 21.608 | 34.362 |
| 12 | -4.0 | 27.736 | 39.149 |
| 13 | -4.0 | 22.505 | 34.853 |
| 14 | -4.0 | 26.843 | 38.361 |
| 15 | -4.0 | 25.793 | 40.063 |
| 16 | -4.0 | 23.559 | 34.963 |
| 17 | -4.0 | 27.826 | 39.364 |
| 18 | -4.0 | 21.972 | 32.727 |
| 19 | -4.0 | 46.216 | 56.701 |
| 20 | -4.0 | 27.969 | 39.227 |

**Supplementary Table 5: Binding modes having interactions between Lys63 and/or Asp65 residues of CD147 receptor with crystallographic water molecules and closed state SARS-CoV-2 ligand.**

| **Mode** | **Free Binding Energy (kcal/mol)** | **Polar interaction distance (Å)** | **Interacting residues** | |
| --- | --- | --- | --- | --- |
|  |  |  | **CD147** | **SARS-CoV-2** |
| 2 | -4.3 | 3.38 | Asp65 in Chain B | R63 |
|  |  | 3.37 and 4.62 | Asp65 in Chain C | S50 |
|  |  | 3.33 | Asp65 in Chain C | R53 |
|  |  | 3.31 | Asp65 in Chain B | H50 |
|  |  | 3.22 | Lys63 in Chain C | R53 |
|  |  | 2.93 | Lys63 in Chain B | R63 |
|  |  | 2.88 | Lys63 in Chain C | G54 |
| 4 | -4.3 | 3.35 | Lys63 in Chain B | Q71 |
|  |  | 3.09 | Lys63 in Chain B | D59 |
| 9 | -4.2 | 3.29 | Asp65 in Chain B | P52 |
|  |  | 3.22 | Asp65 in Chain C | S50 |
|  |  | 3.22 | Lys63 in Chain B | P52 |
|  |  | 3.13 | Lys63 in Chain C | S50 |
|  |  | 3.11 | Lys63 in Chain B | L58 |
|  |  | 3.06 | Lys63 in Chain C | V55 |
|  |  | 2.95 | Asp65 in Chain B | L58 |
| 10 | -4.1 | 3.52 | Asp65 in Chain B | H68 |
|  |  | 3.18 | Asp65 in Chain B | S69 |
|  |  | 3.18 | Asp65 in Chain B | T70 |
|  |  | 2.93 | Lys63 in Chain B | T70 |
| 11 | -4.1 | 4.78 | Asp65 in Chain B | L58 |
|  |  | 3.44 | Asp65 in Chain C | S50 |
|  |  | 3.39 | Asp65 in Chain B | S54 |
|  |  | 3.23 | Asp65 in Chain B | L58 |
|  |  | 3.13 | Lys63 in Chain C | S50 |
|  |  | 3.13 | Lys63 in Chain B | L58 |
|  |  | 3.07 | Lys63 in Chain C | V55 |
| 12 | -4.0 | 3.47 | Asp65 in Chain C | G54 |
|  |  | 3.30 | Asp65 in Chain C | R53 |
|  |  | 3.27 | Asp65 in Chain B | H68 |
|  |  | 3.24 | Lys63 in Chain B | R63 |
|  |  | 3.09 | Asp65 in Chain B | S52 |
| 13 | -4.0 | 3.38 | Lys63 in Chain C | N49 |
|  |  | 3.15 | Asp65 in Chain B | Y48 |
|  |  | 2.97 | Lys63 in Chain C | G54 |
|  |  | 2.77 | Asp65in Chain B | L58 |
| 16 | -4.0 | 3.31 | Asp65 in Chain B | S54 |
|  |  | 3.27 | Lys63 in Chain B | L58 |
|  |  | 3.22 | Asp65 in Chain B | L58 |
|  |  | 3.17 | Lys63 in Chain C | G54 |
|  |  | 3.16 | Lys63 in Chain C | V55 |
| 17 | -4.0 | 3.47 | Asp65 in Chain C | R53 |
|  |  | 3.36 | Asp65 in Chain C | T49 |
|  |  | 3.35 | Lys63 in Chain C | R53 |
|  |  | 3.07 | Lys63 in Chain B | R63 |
| 20 | -4.0 | 3.52 | Asp65 in Chain C | R53 |
|  |  | 3.47 | Asp65 in Chain C | T49 |
|  |  | 3.42 | Asp65 in Chain B | R63 |
|  |  | 3.26 | Asp65 in Chain C | R53 |
|  |  | 3.25 | Lys63 in Chain C | R53 |
|  |  | 3.21 | Asp65 in Chain B | S52 |
|  |  | 3.16 | Lys63 in Chain B | R63 |

**Supplementary Table 6: Twenty best docking scores (lowest binding energy) in docking experiments between CD147 receptor without crystallographic water molecules and closed state SARS-CoV-2 ligand.**

| **Mode** | **Free Binding Energy (kcal/mol)** | **Distance from RMSD lower bound** | **Distance from RMSD upper bound** |
| --- | --- | --- | --- |
| 1 | -3.9 | 0 | 0 |
| 2 | -3.9 | 2.798 | 6.127 |
| 3 | -3.9 | 1.599 | 2.317 |
| 4 | -3.9 | 18.791 | 29.154 |
| 5 | -3.8 | 18.597 | 29.100 |
| 6 | -3.8 | 17.900 | 28.383 |
| 7 | -3.7 | 18.734 | 29.145 |
| 8 | -3.7 | 18.766 | 29.201 |
| 9 | -3.7 | 26.024 | 38.712 |
| 10 | -3.7 | 18.565 | 28.991 |
| 11 | -3.7 | 21.700 | 33.665 |
| 12 | -3.7 | 24.436 | 37.753 |
| 13 | -3.7 | 16.610 | 30.204 |
| 14 | -3.7 | 2.710 | 5.970 |
| 15 | -3.6 | 27.762 | 40.092 |
| 16 | -3.6 | 24.348 | 37.437 |
| 17 | -3.6 | 6.899 | 18.731 |
| 18 | -3.5 | 22.826 | 32.570 |
| 19 | -3.5 | 24.851 | 37.072 |
| 20 | -3.4 | 6.912 | 20.172 |

**Supplementary Table 7: Binding modes having interactions between Lys63 and/or Asp65 residues of CD147 receptor without crystallographic water molecules and closed state SARS-CoV-2 ligand.**

| **Mode** | **Free Binding Energy (kcal/mol)** | **Polar interaction distance (Å)** | **Interacting residues** | |
| --- | --- | --- | --- | --- |
|  |  |  | **CD147** | **SARS-CoV-2** |
| 11 | -3.7 | 3.41 | Lys63 in Chain A | W83 |
|  |  | 3.15 | Asp65 in Chain A | W83 |

**Supplementary Table 8: Delta and Omicron mutations in SARS-CoV-2 do not fall on the region where SARS-CoV-2 closed form binds to Lys63 and Asp65 in CD147, (Modes without the critical residues are not included in the table). Source for mutant variants:** [**https://www.cdc.gov/coronavirus/2019-ncov/variants/variant-info.html**](https://www.cdc.gov/coronavirus/2019-ncov/variants/variant-info.html)

| **Residues in SARS-CoV-2 in PyMoL codes** | | | **Mutations in SARS-CoV-2** |
| --- | --- | --- | --- |
| **Binding mode #** | | **Binding residues** | Delta and Omicron variants |
| 2 | | .268/S50,R53,G54 |  |
| 4 | | .268/Q70; .647/D60 |  |
| 9 | | .218/S50; .268/V55; .269/P52; .270/L58 |  |
| 10 | | .221/T70; .226/S69, T70; .227/H68 |  |
| 11 | | .218/S50, S54; .268/V55 |  |
| 12 | | .218/S52; .226/G54; .268/R53, R63, H68 |  |
| 13 | | .190/Y48; .268/N49, G54; .270/L58 |  |
| 16 | | .218/S54; .268/G54, V55; .270/L58 |  |
| 17 | | .221/T49; .268/R53, R63 |  |
| 20 | | .218/S52; .221T49; .229/R53; .268/R53, R63 |  |
| **MD results having polar interaction pairs at distance 5 Å** | **8 nanoseconds** | Asp65-Lys195, Asp65-Ile197, Asp65-Arg273, Lys63-Gln271, Lys63-Pro272, Lys63-Asp985 |  |
|  | **20 nanoseconds** | Asp65-Arg273, Asp65-Ala292, Lys63-Gln271, Lys63-Trp353, Lys63-Asp985 |  |
|  | **50 nanoseconds** | Asp65-Gln271 (3.12 Å , 3.25 Å), Asp65-Asp88 (3.17 Å), Lys63-Asp985 (2.63 Å), Lys63-Pro986 (4.91 Å), Lys63-Pro987 (2.03 Å) |  |
| **MD results having polar interaction pairs at distance 3 Å** | **8 nanoseconds** | Asp65-Lys195, Asp65-Arg273, Lys63-Asp985 |  |
|  | **20 nanoseconds** | Asp65-Arg273 |  |
|  | **50 nanoseconds** | Lys63-Pro987 (2.03 Å) |  |

**Supplementary Table 9: Twenty best docking scores (lowest binding energy) in docking experiments between retinal specific CD147 receptor with crystallographic water molecules and open state SARS-CoV-2 ligand.**

| **Mode** | **Free Binding Energy (kcal/mol)** | **Distance from RMSD lower bound** | **Distance from RMSD upper bound** |
| --- | --- | --- | --- |
| 1 | -4.9 | 0 | 0 |
| 2 | -4.6 | 2.096 | 3.362 |
| 3 | -4.5 | 2.189 | 3.924 |
| 4 | -4.4 | 25.412 | 37.492 |
| 5 | -4.4 | 25.700 | 36.810 |
| 6 | -4.4 | 3.209 | 11.636 |
| 7 | -4.4 | 1.837 | 2.878 |
| 8 | -4.3 | 25.711 | 36.617 |
| 9 | -4.3 | 24.508 | 37.453 |
| 10 | -4.3 | 3.587 | 11.762 |
| 11 | -4.2 | 25.279 | 37.551 |
| 12 | -4.1 | 5.495 | 21.759 |
| 13 | -4.1 | 11.808 | 25.779 |
| 14 | -4.1 | 25.286 | 37.946 |
| 15 | -4.1 | 25.907 | 38.488 |
| 16 | -4.1 | 15.615 | 33.543 |
| 17 | -4.1 | 23.841 | 37.167 |
| 18 | -4.1 | 2.843 | 11.006 |
| 19 | -4.0 | 3.352 | 11.329 |
| 20 | -4.0 | 25.342 | 36.933 |

**Supplementary Table 10: Twenty best docking scores (lowest binding energy) in docking experiments between retinal specific CD147 receptor without crystallographic water molecules and open state SARS-CoV-2 ligand.**

| **Mode** | **Free Binding Energy (kcal/mol)** | **Distance from RMSD lower bound** | **Distance from RMSD upper bound** |
| --- | --- | --- | --- |
| 1 | -3.8 | 0 | 0 |
| 2 | -3.6 | 1.540 | 1.540 |
| 3 | -3.3 | 2.037 | 3.175 |
| 4 | -3.3 | 1.637 | 2.387 |
| 5 | -3.1 | 5.155 | 15.132 |
| 6 | -3.1 | 3.855 | 16.389 |
| 7 | -3.0 | 2.681 | 4.909 |
| 8 | -2.8 | 9.763 | 19.631 |
| 9 | -2.8 | 4.342 | 16.816 |
| 10 | -2.7 | 2.685 | 5.148 |
| 11 | -2.6 | 4.287 | 17.005 |
| 12 | -2.6 | 10.267 | 20.174 |
| 13 | -2.6 | 2.889 | 5.577 |
| 14 | -2.6 | 4.546 | 16.600 |
| 15 | -2.5 | 4.446 | 21.005 |
| 16 | -2.5 | 9.849 | 20.125 |
| 17 | -2.5 | 3.876 | 17.444 |
| 18 | -2.4 | 7.120 | 22.014 |
| 19 | -2.3 | 6.629 | 21.190 |
| 20 | -2.2 | 4.765 | 20.940 |

**Supplementary Table 11: Twenty best docking scores (lowest binding energy) in docking experiments between retinal specific CD147 receptor with crystallographic water molecules and closed state SARS-CoV-2 ligand.**

| **Mode** | **Free Binding Energy (kcal/mol)** | **Distance from RMSD lower bound** | **Distance from RMSD upper bound** |
| --- | --- | --- | --- |
| 1 | -4.6 | 0 | 0 |
| 2 | -4.6 | 1.380 | 2.111 |
| 3 | -4.6 | 5.914 | 15.926 |
| 4 | -4.5 | 5.377 | 16.059 |
| 5 | -4.4 | 2.159 | 4.033 |
| 6 | -4.4 | 11.040 | 21.601 |
| 7 | -4.4 | 5.066 | 15.764 |
| 8 | -4.4 | 2.334 | 4.312 |
| 9 | -4.4 | 5.126 | 15.902 |
| 10 | -4.3 | 5.143 | 15.401 |
| 11 | -4.3 | 5.176 | 15.698 |
| 12 | -4.3 | 22.483 | 34.368 |
| 13 | -4.3 | 5.072 | 14.840 |
| 14 | -4.3 | 27.769 | 37.991 |
| 15 | -4.2 | 4.979 | 15.049 |
| 16 | -4.2 | 27.713 | 37.944 |
| 17 | -4.1 | 5.174 | 14.890 |
| 18 | -4.1 | 3.494 | 13.747 |
| 19 | -4.1 | 4.141 | 16.173 |
| 20 | -4.1 | 2.315 | 4.215 |

**Supplementary Table 12: Twenty best docking scores (lowest binding energy) in docking experiments between retinal specific CD147 receptor without crystallographic water molecules and closed state SARS-CoV-2 ligand.**

| **Mode** | **Free Binding Energy (kcal/mol)** | **Distance from RMSD lower bound** | **Distance from RMSD upper bound** |
| --- | --- | --- | --- |
| 1 | -3.8 | 0 | 0 |
| 2 | -3.6 | 1.540 | 2.145 |
| 3 | -3.3 | 2.037 | 3.175 |
| 4 | -3.3 | 1.637 | 2.387 |
| 5 | -3.1 | 5.155 | 15.132 |
| 6 | -3.1 | 3.855 | 16.389 |
| 7 | -3.0 | 2.681 | 4.909 |
| 8 | -2.8 | 9.763 | 19.631 |
| 9 | -2.8 | 4.342 | 16.816 |
| 10 | -2.7 | 2.685 | 5.148 |
| 11 | -2.6 | 4.287 | 17.005 |
| 12 | -2.6 | 10.267 | 20.174 |
| 13 | -2.6 | 2.889 | 5.577 |
| 14 | -2.6 | 4.546 | 16.600 |
| 15 | -2.5 | 4.446 | 21.005 |
| 16 | -2.5 | 9.849 | 20.125 |
| 17 | -2.5 | 3.876 | 17.444 |
| 18 | -2.4 | 7.120 | 22.014 |
| 19 | -2.3 | 6.629 | 21.190 |
| 20 | -2.2 | 4.765 | 20.940 |
